# The first evidence of global meat phosphoproteome changes in response to pre-slaughter stress

**DOI:** 10.1186/s12864-019-5943-3

**Published:** 2019-07-17

**Authors:** Ariadna Mato, Raquel Rodríguez-Vázquez, María López-Pedrouso, Susana Bravo, Daniel Franco, Carlos Zapata

**Affiliations:** 1 Department of Zoology, Genetics and Physical Anthropology, University of Santiago de Compostela, 15782 Santiago de Compostela, Spain; 2 Proteomics Laboratory, CHUS, 15782 Santiago de Compostela, Spain; 3 Meat Technology Center of Galicia, 32900 Ourense, Spain

**Keywords:** *Bos taurus* - DFD meat - meat phosphoproteome, Beef quality, Meat tenderness, Pre-slaughter stress biomarkers, *Post-mortem* metabolism

## Abstract

**Background:**

Pre-slaughter stress (PSS) impairs animal welfare and meat quality. Dark, firm and dry (DFD) are terms used to designate poor quality meats induced by PSS. Protein phosphorylation can be a potentially significant mechanism to explain rapid and multiple physiological and biochemical changes linked to PSS-dependent muscle-to-meat conversion. However, the role of reversible phosphorylation in the response to PSS is still little known. In this study, we report a comparative phosphoproteomic analysis of DFD and normal meats at 24 h *post*-*mortem* from the *longissimus thoracis* (LT) bovine muscle of male calves of the Rubia Gallega breed. For this purpose, two-dimensional gel electrophoresis (2-DE), in-gel multiplex identification of phosphoproteins with PRO-Q Diamond phosphoprotein-specific stain, tandem (MALDI-TOF/TOF) mass spectrometry (MS), novel quantitative phosphoproteomic statistics and bioinformatic tools were used.

**Results:**

Noticeable and statistically significant differences in the extent of protein phosphorylation were detected between sample groups at the qualitative and quantitative levels. Overall phosphorylation rates across significantly changed phosphoproteins were about three times higher in DFD than in normal meat. Significantly changed phosphoproteins involved a variable number of isoforms of 13 myofibrillar and sarcoplasmic nonredundant proteins. However, fast skeletal myosin light chain 2 followed by troponin T, F-actin-capping and small heat shock proteins showed the greatest phosphorylation change, and therefore they were the most important phosphoproteins underlying LT muscle conversion to DFD meat in the Rubia Gallega breed.

**Conclusions:**

This is the first study reporting global meat phosphoproteome changes in response to PSS. The results show that reversible phosphorylation is a relevant mechanism underlying PSS response and downstream effects on meat quality. This research opens up novel horizons to unravel the complex molecular puzzle underlying muscle-to-meat conversion in response to PSS.

**Electronic supplementary material:**

The online version of this article (10.1186/s12864-019-5943-3) contains supplementary material, which is available to authorized users.

## Background

Phosphorylation is a ubiquitous protein post-translational modification that regulates a plethora of fundamental cell processes such as signal transduction pathways, cell cycle and apoptosis [1]. Reversible phosphorylation by the concerted action of a complex network of protein kinases and protein phosphatases plays a key regulatory role in the biochemical processes underlying muscle contraction and metabolism during the *post*-*mortem* muscle-to-meat conversion [2–5]. *Post*-*mortem* changes in the phosphorylation status of myofibrillar proteins and glycolytic enzymes in bovine, ovine and porcine muscles have been linked to differences in the meat quality traits of tenderness and color stability [2, 4, 6–8]. The available evidence suggests that reversible phosphorylation of proteins involved in muscle contraction and glycolysis can influence meat quality due to its *post*-*mortem* effects on pH decline and the development of *rigor mortis* [4, 9–11].

Stress is a key factor for animal welfare that influences meat quality traits [12, 13]. PSS can be elicited by multiple factors such as physical and psychological stressors linked to transport and handling activities from farms to abattoirs [14–17]. Physical and psychological stressors that trigger PSS response include but are not limited to environmental temperature, human presence, unfamiliar environments, mixing of animals from different social group, water and feed deprivation during transportation, loading and unloading practices and lairage in slaughter house [17–20]. The strength of PSS depends on the type, intensity and duration of stressors. PSS has been classified in acute or short stress when the duration of transport does not exceed a few days and chronic stress whether the transport lasts longer [17]. PSS can also be modulated by previous experiences and acquired learning, endogenous animal factors (e.g. genotype, age, sex) as well as by individual psychological and physiological state [21, 22]. Stress response to stressors is typically initiated through the activation of the autonomic nervous system (ANS) and the hypothalamic-pituitary-adrenal (HPA) axis mediated by catecholamines and glucocorticoids [21, 23, 24]. Neuroendocrine systems trigger a wide range of physiological and biochemical changes that affect the animal welfare and influence key processes when muscle turns into meat [12, 13, 25]. PSS can produce poor quality meats classified as DFD and pale, soft and exudative (PSE) meats [25]. They are therefore an excellent model to extent our knowledge about the biochemical processes underlying stress response and downstream effects on meat quality traits.

PSS can induce the *ante*-*mortem* depletion of glycogen reserves in bovine muscle which are used as source of energy to supply ATP for muscle contraction and relaxation [20, 26–28]. Glycogen-depleted muscle fibers in the immediate *post*-*mortem* period generate low amounts of lactic acid through anaerobic glycolysis which alters the normal process of meat acidification. In DFD meat, pH values at 12–48 h *post*-*mortem* are higher than 6.0, while in normal meat the corresponding pH falls to values of about 5.4–5.7 [25, 27]. Higher pH values can have a detrimental impact on the meat general appearance and many other determinants of meat quality. In comparison to normal meat, DFD condition is characterized by a darker color, a superficially drier and firmer texture, higher water-holding capacity (WHC) with little or no exudates, less protein denaturation, inferior taste, more susceptibility to microbial growth and a high potential of spoilage at an early meat aging [25–27, 29–35]. It must be highlighted, however, that DFD meat is usually tenderer than normal meat [20, 29, 32, 35]. Overall, it is a type of meat with lower consumer acceptability [36] that causes significant economic losses to the beef industry [20–25].

Proteomics has successfully contributed to unraveling the biochemical processes determining meat quality variations in response to varied stress inducers [37, 38]. To our knowledge, only a few recent studies have reported global proteome changes in DFD and PSE meat of cattle and broiler, respectively [35, 39, 40]. On the other hand, phosphoproteome studies have showed that two pre-slaughter stressors (i.e. transport and lairage) had no apparent effect on the global protein phosphorylation in lamb meat at 24 h *post*-*mortem*, as well as no differences in the phosphorylation levels of pork myofibrillar proteins between PSE and normal meats at early *post-mortem* time [41, 42]. In contrast, highly phosphorylated fast skeletal myosin regulatory light chain 2 (MYLPF) isoforms showed the most intense relative change across the proteome between DFD and normal meats from LT bovine muscle [35]. This finding suggests that reversible phosphorylation could be a significant mechanism in response to PSS. It is noteworthy that PSS triggers multiple physiological and biochemical changes in beef muscle in very short intervals of time. Accordingly, reversible phosphorylation has the potential to rapidly alter regulatory processes associated with muscle conversion into meat. In this regard, phosphoproteome changes have been reported in pigs with Halothane gene mutations and anomalous muscle energy metabolism that produce PSE meat [43].

This study aimed to assess for the first time the phosphoproteome differences between DFD and normal bovine meat. For this purpose, the phosphoproteome profiles of DFD and unaffected control meat from LT bovine muscle of the Rubia Gallega breed were compared using previously characterized meat samples at the proteome level. It will allow us to evaluate whether reversible phosphorylation plays a significant role in response to PSS with outstanding effects downstream on muscle-to-meat conversion processes.

## Results

### Phosphoproteome profiles of DFD and control meat samples by 2-DE

**Fig. 1:**
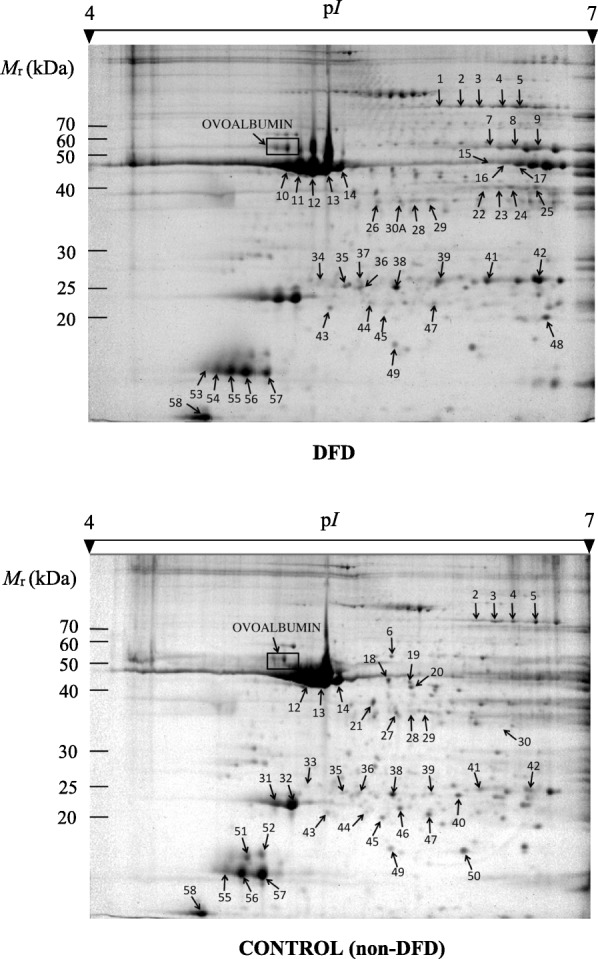
Representative 2-DE gel profiles of DFD (above) and control (below) meat samples from the LT bovine muscle stained with Pro-Q Diamond and subsequently with SYPRO Ruby. Phosphoprotein spots with statistically significant qualitative (presence/absence) and quantitative (changes in intensity) differential phosphorylation are marked and numbered. Numbered spots were excised from gels for phosphoprotein identification by MALDI-TOF and MALDI-TOF/TOF MS.

Figure 1 shows 2-DE representative proteomic profiles of DFD and control (non-DFD) meat samples at 24 h *post*-*mortem* derived from LT muscle on gels stained with phosphoprotein-specific Pro-Q Diamond stain and post-stained with non-specific SYPRO Ruby stain. PeppermintStick markers showed the specificity of Pro-Q Diamond for phosphoproteins under our experimental protocols. We found that DFD and normal meats exhibited markedly differentiated phosphoproteome profiles at the qualitative and quantitative levels. First of all, the percentage of Pro-Q-Diamond-stained reproducible spots was similar in DFD (14.6%, 46 out of 314 spots) and control (13.3%, 41 out of 308 spots) meat (*P*-value = 0.76, two-tailed Fisher’s exact test). Nevertheless, only 28 Pro-Q-Diamond-stained spots were shared between sample groups. Therefore, 18 and 13 spots were unique spots with phosphorylation signal only in DFD and control meats, respectively.

Quantitative estimates of phosphorylation levels over 2-DE spots in DFD and control samples were assessed by the phosphorylation rate (*PR*) statistic (Additional file 1: Table S1). In total, 54.2% (32 out of 59) of spots showed statistically significant differences in the *PR* mean value between sample groups by using 95% bootstrap CIs obtained by the bias-corrected percentile method and adjusted with the Bonferroni correction. Significantly changed phosphoprotein spots are shown in Table 1. Overall, phosphorylation levels were far higher in DFD meat than in control meat: *PR*_*DFD*_ and *PR*_*C*_ values across significant cases averaged (±SE, standard error) 0.33 ± 0.06 and 0.13 ± 0.03, respectively (*P* < 0.05, two-tailed Mann-Whitney test). It is also important to note that most (97%) significant cases were unique spots in either DFD or control meats.

**Table 1:** Significantly (*P* < 0.05) changed phosphoprotein spots between DFD and control (non DFD) meat samples of LT bovine muscle

Spot no.^a^	*PR*_*DFD*_		*PR*_*C*_	
	Mean (±SE)	Adjusted 95% CIs (CL, CU)^b^	Mean (±SE)	Adjusted 95% CIs (CL, CU)
1	0.41 ± 0.10	0.292, 0.603	0	N/A
5	0.08 ± 0.03	0.051, 0.108	0.36 ± 0.12	0.169, 0.696
6	0	N/A	0.12 ± 0.06	0.060, 0.171
7	0.54 ± 0.21	0.206, 0.939	0	N/A
8	0.48 ± 0.08	0.281, 0.614	0	N/A
9	0.36 ± 0.10	0.153, 0.630	0	N/A
10	0.33 ± 0.06	0.205, 0.435	0	N/A
11	0.31 ± 0.03	0.238, 0.371	0	N/A
15	0.35 ± 0.16	0.154, 0.655	0	N/A
16	0.31 ± 0.04	0.237, 0.379	0	N/A
18	0	N/A	0.12 ± 0.04	0.067, 0.186
19	0	N/A	0.15 ± 0.05	0.041, 0.206
20	0	N/A	0.13 ± 0.02	0.105, 0.179
21	0	N/A	0.11 ± 0.05	0.031, 0.187
22	0.70 ± 0.12	0.458, 0.948	0	N/A
23	0.69 ± 0.08	0.505, 0.874	0	N/A
24	0.66 ± 0.10	0.452, 0.804	0	N/A
25	0.63 ± 0.06	0.447, 0.693	0	N/A
26	0.72 ± 0.19	0.531, 0.904	0	N/A
27	0	N/A	0.37 ± 0.19	0.175, 0.563
30	0	N/A	0.73 ± 0.19	0.358, 0.929
30A	0.71 ± 0.15	0.402, 0.879	0	N/A
33	0	N/A	0.49 ± 0.24	0.033, 0.839
34	0.69 ± 0.25	0.192, 0.951	0	N/A
37	0.65 ± 0.19	0.467, 0.835	0	N/A
44	0	N/A	0.46 ± 0.20	0.087, 0.776
46	0	N/A	0.13 ± 0.03	0.073, 0,177
50	0	N/A	0.09 ± 0.05	0.011, 0.194
51	0	N/A	0.38 ± 0.14	0.211, 0.664
52	0	N/A	0.37 ± 0.19	0.020, 0.764
53	0.93 ± 0.05	0.831, 1.000	0	N/A
54	0.94 ± 0.04	0.875, 1.000	0	N/A

^a^Gel position of assigned spots is shown in Fig. 1. ^b^Simultaneous non-parametric bootstrap CIs (CL, lower bound; CU, upper bound) determined by the bias-corrected percentile method (10,000 replicates) and adjusted by the Bonferroni method. N/A, not applicable

### Phosphoprotein identification by tandem MS

Phosphoprotein spots with significantly changed phosphorylation level in sample groups (i.e. 32 spots) were excised from 2-DE gels, processed for in-gel trypsin digestion and confidently (*P* < 0.05) identified by MALDI-TOF and MALDI-TOF/TOF MS. The resulting protein identifications are shown in Table 2 (see Additional file 1: Table S2 for further information). It can be seen that 13 nonredundant proteins with a variable number of isoforms (zero to seven) were identified. Protein identifications corresponded to myofibrillar proteins [actin (ACTA1), fast skeletal myosin regulatory light chain 2 (MYLPF), myosin regulatory light chain 2 (MYL2) and myosin, light chain 6B (MYL6B)]; muscle contraction regulation proteins [slow skeletal muscle troponin T (TNNT1) and fast skeletal muscle troponin T (TNNT3)]; actin polymerization protein [alpha-2 subunit of the F-actin-capping protein (CAPZA2)]; enzymes involved in glycogenolysis (phosphoglucomutase-1, PGM1), glycolysis (beta-enolase, ENO3) and interconversion between creatine and phosphocreatine (creatin kinase M-type, CKM); cytochrome b-c1 complex subunit (UQCRC1); and small heat shock proteins beta-1 (HSPB1) and beta-6 (HSPB6).

**Table 2:** Identification of differentially (*P* < 0.05) phosphorylated 2-DE protein spots in DFD and control meat samples by MALDI-TOF and MALDI-TOF/TOF MS

Spot no.^a^	Type of meat	Protein identity^b^	Abbrev. (isospot)	Accession	*M*_r_ (kDa) Obs./Exp^c^	p*I* Obs./Exp^c^	Score^d^	Sequence coverage^e^
1	DFD	Phosphoglucomutase-1	PGM1 (1)	PGM1_BOVIN	66.2/61.8	5.90/6.36	415	45
5	DFD	Unidentified			69.9/61.8	6.98/6.36		
	Control	Phosphoglucomutase-1	PGM1 (2)	PGM1_BOVIN	69.9/61.8	6.98/6.36	552	41
6	Control	Cytochrome b-c1 complex subunit 1, mitocondrial	UQCRC1	QCR1_BOVIN	53.9/53.4	5.60/5.94	364	47
7	DFD	Beta-enolase	ENO3 (1)	ENOB_BOVIN	51.9/47.4	6.33/7.60	269	39
8	DFD	Beta-enolase	ENO3 (2)	ENOB_BOVIN	51.8/47.4	6.45/7.60	253	34
9	DFD	Beta-enolase	ENO3 (3)	ENOB_BOVIN	51.7/47.4	6.63/7.60	269	48
10	DFD	Actin, alpha skeletal muscle	ACTA1 (1)	ACTS_BOVIN	42.2/42.4	4.95/5.23	474	62
11	DFD	Actin, alpha 1, skeletal muscle	ACTA1 (2)	A4IFM8_BOVIN	41.6/42.4	4.99/5.23	439	51
15	DFD	Creatin kinase M-type	CKM (1)	KCRM_BOVIN	45.0/43.2	6.34/6.63	266	39
16	DFD	Creatin kinase M-type	CKM (2)	KCRM_BOVIN	44.1/43.1	6.42/6.63	369	49
18	Control	Actin, alpha, skeletal muscle	ACTA1 (3)	ACTS_BOVIN	45.3/42.4	5.58/5.23	519	43
19	Control	Actin, alpha, skeletal muscle	ACTA1 (4)	ACTS_BOVIN	44.5/42.4	5.78/5.23	453	39
20	Control	Actin, alpha, skeletal muscle	ACTA1 (5)	ACTS_BOVIN	40.0/42.4	5.80/5.23	423	33
21	Control	Actin, alpha, skeletal muscle	ACTA1 (6)	ACTS_BOVIN	36.5/42.4	5.45/5.23	484	53
22	DFD	Troponin T, fast skeletal muscle	TNNT3 (1)	TNNT3_BOVIN	37.8/32.1	6.31/5.99	66	8
23	DFD	Troponin T fast skeletal muscle type	TNNT3 (2)	TNNT3_BOVIN	37.8/32.1	6.38/5.59	62	13
24	DFD	Troponin T, fast skeletal muscle	TNNT3 (3)	TNNT3_BOVIN	37.8/32.1	6.44/5.99	145	13
25	DFD	Troponin T, fast skeletal muscle	TNNT3 (4)	TNNT3_BOVIN	37.8/32.1	6.62/5.99	60	13
26	DFD	F-actin-capping protein subunit alpha-2	CAPZA2	CAZA2_BOVIN	35.4/33.1	5.48/5.57	74	16
27	Control	Actin, alpha skeletal muscle	ACTA1 (7)	ACTS_BOVIN	35.8/42.4	5.54/5.23	300	23
30	Control	Troponin T, slow skeletal muscle	TNNT1 (1)	TNNT1_BOVIN	33.8/31.3	6.53/5.71	109	20
30A	DFD	Troponin T, slow skeletal muscle	TNNT1 (2)	TNNT1_BOVIN	35.5/31.3	5.63/5.71	71	14
33	Control	Heat shock protein beta-1	HSPB1 (1)	HSPB1_BOVIN	25.8/22.4	5.05/5.98	230	36
34	DFD	Heat shock protein beta-1	HSPB1 (2)	E1BEL7_BOVIN	26.2/22.6	5.09/5.77	159	20
37	DFD	Heat shock protein beta-1	HSPB1 (3)	E1BEL7_BOVIN	25.9/22.6	5.34/5.77	163	27
44	Control	Myosin, light chain 6B, alkali, smooth muscle and non-muscle	MYL6B (1)	Q148H2_BOVIN	20.9/23.5	5.43/5.40	109	26
46	Control	Myosin, light chain 6B, alkali, smooth muscle and non-muscle	MYL6B (2)	Q148H2_BOVIN	21.1/23.5	5.69/5.40	329	58
50	Control	Heat shock protein beta-6	HSPB6	HSPB6_BOVIN	18.7/17.5	6.28/5.95	146	39
51	Control	Myosin regulatory light chain 2, ventricular/cardiac muscle isoform	MYL2 (1)	MLRV_BOVIN	18.1/18.9	4.86/4.86	399	65
52	Control	Myosin regulatory light chain 2, ventricular/cardiac muscle isoform	MYL2 (2)	F1ME15_BOVIN	18.2/18.9	4.90/4.86	380	76
53	DFD	Myosin regulatory light chain 2, fast skeletal muscle isoform	MYLPF (1)	MLRS_BOVIN	17.7/19.1	4.74/4.91	363	61
54	DFD	Myosin regulatory light chain 2, fast skeletal muscle isoform	MYLPF (2)	MLRS_BOVIN	17.6/19.1	4.77/4.91	88	23

^a^Gel position of assigned spots is shown in Fig. 1. ^b^All identified proteins were matched to *Bos taurus* proteins. ^c^Theoretical (Th) isoelectric point (p*I*) and molecular mass (*M*_r_) were obtained from UniProtKB/Swiss-Prot databases. Observed (Ob) p*I* and *M*_r_ were obtained from the spot position on the gel. ^d^The Mascot baseline statistically significant (*P*-value < 0.05) score was 56. ^e^Percentage of coverage of the entire amino acid sequence by matched peptides

### Quantitation of protein phosphorylation changes

**Fig. 2:**
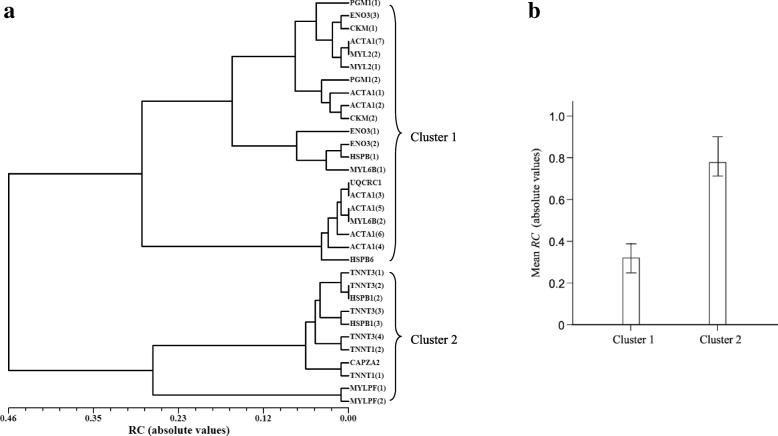
Cluster analysis of phosphoproteins with significant (*P*-value < 0.05) differential phosphorylation level in DFD and control (non-DFD) meats based on *RC*-values. **a** UPGMA dendrogram constructed from the matrix of mean differences in *RC* between pairs of phosphoproteins (in absolute value) using NTSYS software. The two main clusters in the resulting dendrogram were denoted as clusters 1 and 2. **b** Mean values of *RC* (absolute values) for phosphoproteins of clusters 1 and 2 along with their 99% bootstrap CIs

**Fig. 3:**
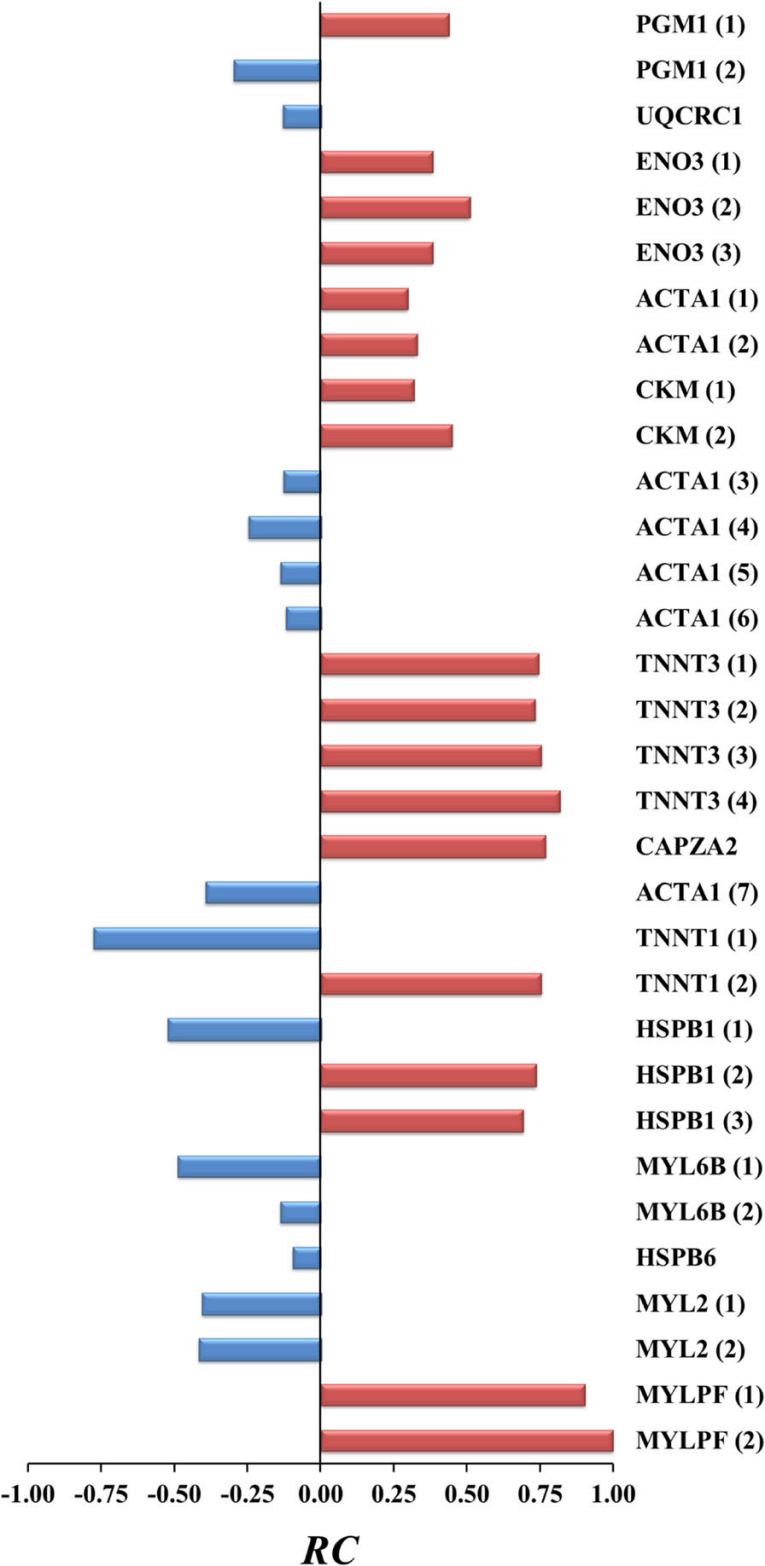
Quantitation of phosphorylation changes (*P* < 0.05) between phosphoproteins of DFD and control meat samples from the LT bovine muscle assessed by the *RC* coefficient. Phosphoproteins in DFD meat with higher (red) and lower (blue) phosphorylation levels than in control (or normal) meat are shown

Quantitative changes of *PR* between DFD and control meats were measured by the fold change (*FC*) and relative change (*RC*) coefficients (Table 3). It can be seen that *FC*, commonly used for measuring changes in protein abundance, was not useful to quantify changes in the phosphorylation status. Thus, most *FC*-values over phosphoproteins were -∞ or + ∞ because of the high presence of unique phosphorylated protein spots in DFD or control meats. Unlike *FC*, the *RC* measure has shown to have advantageous statistical properties in a wide diversity of proteomic scenarios because it always ranges from − 1.0 and + 1.0 across both unshared and shared spots between sample groups [35, 44–46]. Applying *RC*, we found that MYLPF isoforms underwent the strongest quantitative change at the phosphorylation level (*RC*_*MYLPF1*(1)_ = + 0.99; *RC*_*MYLPF1*(2)_ = + 1.0); followed by TNNT1 (1–2), CAPZA2, TNNT3 (1–2) and HSPB1 (2) phosphoproteins with absolute *RC* values higher than 0.70. The UPGMA dendrogram based on pairwise mean differences in *RC* between sample groups (absolute values) distinguished two major phosphoprotein clusters (Fig. 2a). The phosphoprotein cluster including MYLPF (1–2), TNNT1 (1–2), TNNT3 (1–4), HSPB1 (2) and CAPZA2 exhibited statistically significant higher levels of phosphorylation than those of the other cluster (*P* < 0.05, 95% bootstrap CIs adjusted with the Bonferroni correction; Fig. 2b). The sign or direction of the change in phosphorylation levels between DFD and control meats was extremely variable across proteins identified in this study without any apparent function-dependent trend (Fig. 3).

**Table 3:** Change in the *PR* of proteins between DFD and control meats measured by *FC* and *RC* coefficients

Spot no.^a^	Protein (isospot)	*FC*	*RC*	Spot no.	Protein (isospot)	*FC*	*RC*
1	PGM1 (1)	+∞	+ 0.44	24	TNNT3 (3)	+∞	+ 0.70
5	PGM1 (2)	−4.50	−0.30	25	TNNT3 (4)	+∞	+ 0.67
6	UQCRC1	-∞	−0.13	26	CAPZA2	+∞	+ 0.77
7	ENO3 (1)	+∞	+ 0.57	27	ACTA1 (7)	-∞	−0.39
8	ENO3 (2)	+∞	+ 0.51	30	TNNT1 (1)	-∞	−0.78
9	ENO3 (3)	+∞	+ 0.38	30A	TNNT1 (2)	+∞	+ 0.76
10	ACTA1 (1)	+∞	+ 0.35	33	HSPB1 (1)	-∞	−0.52
11	ACTA1 (2)	+∞	+ 0.33	34	HSPB1 (2)	+∞	+ 0.73
15	CKM (1)	+∞	+ 0.37	37	HSPB1 (3)	+∞	+ 0.69
16	CKM (2)	+∞	+ 0.32	44	MYL6B (1)	-∞	−0.49
18	ACTA1 (3)	-∞	−0.13	46	MYL6B (2)	-∞	−0.14
19	ACTA1 (4)	-∞	−0.16	50	HSPB6	-∞	−0.10
20	ACTA1 (5)	-∞	−0.14	51	MYL2 (1)	-∞	−0.40
21	ACTA1 (6)	-∞	−0.12	52	MYL2 (2)	-∞	−0.39
22	TNNT3 (1)	+∞	+ 0.74	53	MYLPF (1)	+∞	+ 0.99
23	TNNT3 (2)	+∞	+ 0.73	54	MYLPF (2)	+∞	+ 1.00

^a^Gel position of assigned spots is shown in Fig. 1

### Functional categorization of phosphoproteins from GO terms

**Fig. 4:**
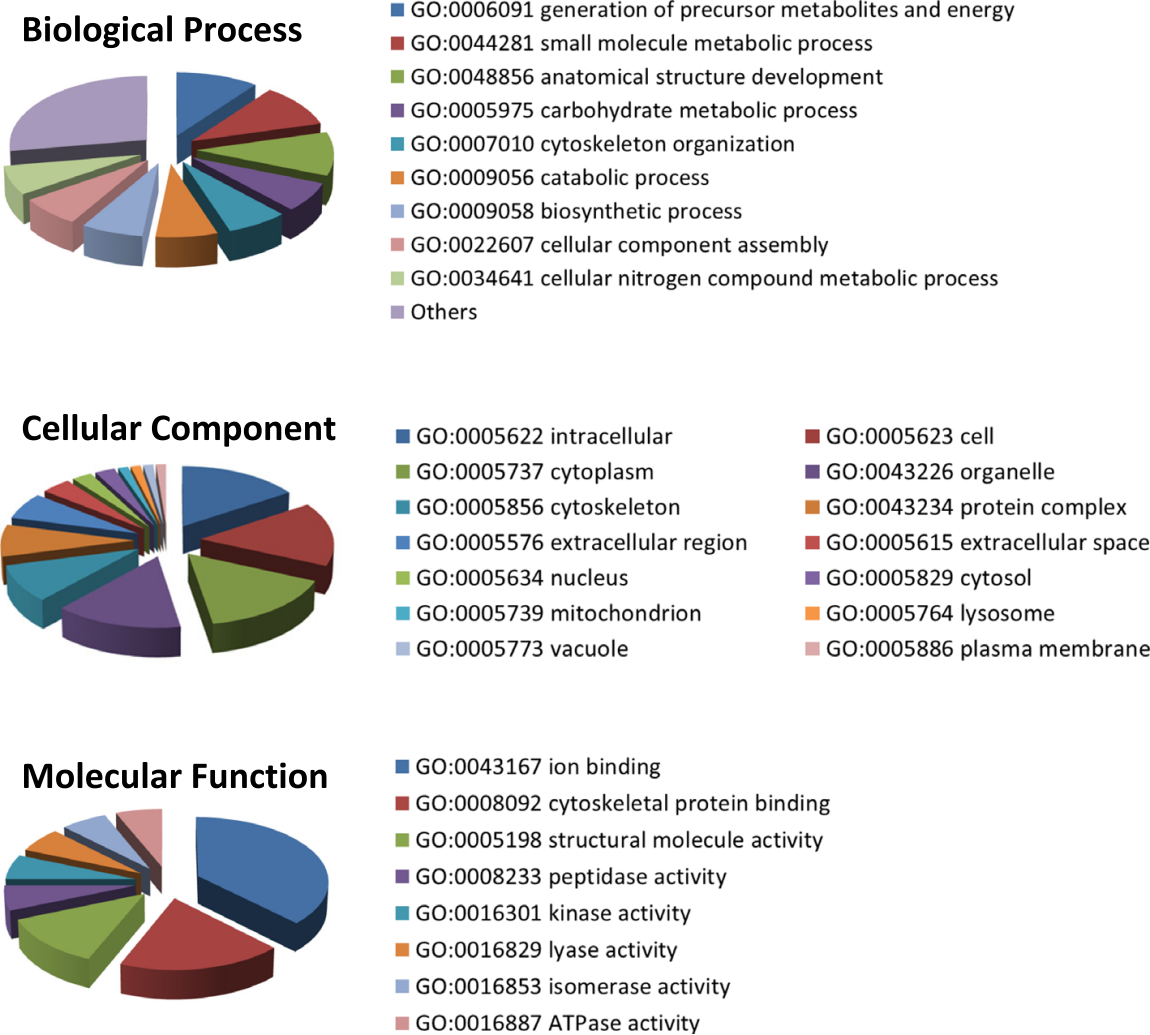
Pie chart reporting the distribution of high level GO functional annotation terms (GO Slim) of the three different ontologies (biological process, molecular function and cellular component) for the 13 differentially phosphorylated proteins between DFD and control meats. GO slim terms were retrieved by mean of the Slimmer tool of AmiGO software

Analysis of broader or high level GO slim terms using the Slimmer tool of AmiGO software as well as fine-grained GO terms by means of QuickGo tool showed that the 13 differentially phosphorylated proteins participate in biological processes such as glycogen biosynthesis and skeletal muscle contraction and activities such as actin, tropomyosin and calcium ion binding. They can be found in different locations such as extracellular space, cell membrane and inside cells as a constituent component of myosin or troponin complex (Fig. 4; Additional file 1: Table S3). In addition, FatiGo enrichment analysis from GO, InterPro and KEGG database terms revealed that three InterPro terms [troponin (IPR001978), alpha crystallin/heat shock protein (IPR001436) and heat shock protein Hsp20 (IPR002068)] and three GO cellular component terms [actin cytoskeleton (GO: 0015629), myofibril (GO: 0030016) and contractile fiber (GO: 0043292)] were significantly (*P* < 0.05) overrepresented in the proteome of *B. taurus* (Additional file 1: Table S4).

### Phosphoprotein-phosphoprotein interaction networks

**Fig. 5:**
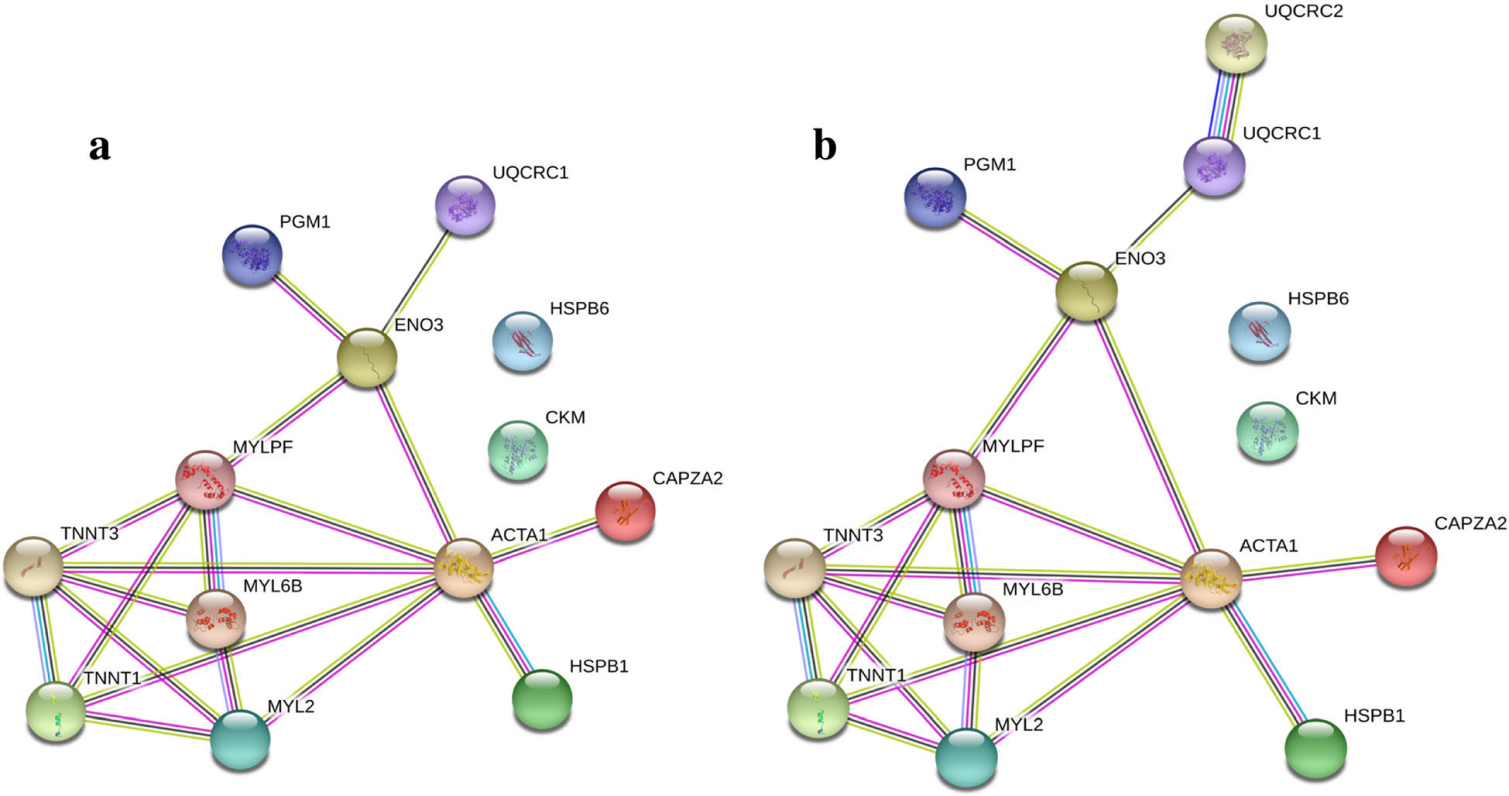
Graphs showing the interaction networks of differentially (*P* < 0.05) phosphorylated proteins in DFD and control meats, according to STRING confidence view. **a)** STRING network view only for differentially phosphorylated proteins identified in the present study (specific settings: number of interaction to show, zero in the first and the second shell). **b**) The same interaction networks adding other directly interacting proteins (specific settings: number of interaction to show, one in the first shell and zero in the second shell). The network nodes (circles) are phosphoproteins and the edges show known or predicted functional associations (threshold: 0.4, medium confidence interval). Colored lines between the phosphoproteins indicate the various types of interaction evidence (blue line: co-occurrence; light blue line: database evidence; black line: coexpression; green line: neighborhood evidence; purple line: experimental evidence; red line: fusion evidence; yellow line: text mining evidence)

The network of known and predicted interactions of proteins with differential (*P* < 0.05) phosphorylation level in DFD and control meats according to STRING database searching is shown in Fig. 5. The interaction network map revealed several outstanding facts. All phosphoproteins were clustered into a single interaction network, with the exception of HSPB6 and CKM (Fig. 5a; settings: zero interactions to show in the first and second shell). Most phosphoprotein-phosphoprotein interactions involved muscle structural-contractile, muscle contraction regulation and actin polymerization functions. ACTA1 showed the largest number of interacting partners, which suggests that it could play a key role in response mechanisms to PSS. In addition, ACTA1 together with MYLPF were the only two nodes on the interaction map connecting structural-contractile muscle phosphoproteins with metabolism phosphoenzymes. It was found that no other protein interacts directly with the phosphoproteins identified in this study (Fig. 5b; settings: number of interaction to show, one in the first shell and none in the second shell). This result suggests that the comparative analysis of 2-DE-based phosphoproteome profiles between DFD and control meats was able to successfully identify the core of protein-protein interaction networks linked to PSS.

Two small clusters of related phosphoproteins were obtained when a phosphoprotein-phosphoprotein co-expression specific network was retrieved from STRING database: a cluster of structural-contractile phosphoproteins (MYLPF and TNNT3) and other group of metabolism phosphoenzymes (PGM1, ENO3 and UQCRC1) (Additional file 1: Figure S1; settings: zero interactions to show in the first and second shell). It is noteworthy, however, that STRING database is based on pre-existing knowledge of protein-protein interactions completed in very diverse biological scenarios. By way of contrast, our observations provide previously unknown information on correlated changes at the phosphorylation level of proteins that occur specifically in response to PSS. Accordingly, we found more extensive correlations between proteins differentially phosphorylated in DFD and control meats than STRING database searching (Table 3, Fig. 2).

## Discussion

Our observations revealed remarkable phosphoproteome changes induced by PSS. Global protein phosphorylation levels were found to be about three times higher in DFD meat than in normal meat and most differentially phosphorylated spots were only identified either in DFD or control samples. In addition, changes in the status of protein phosphorylation between DFD and normal meats were noticeably higher than those changes in protein abundance from the same meat samples previously reported by Franco et al. [35]. Thus, we identified a total of 32 protein spots with statistically significant differences in the phosphorylation status between DFD and normal meats, whereas only 10 protein spots showed statistically significant differential abundance over total proteome (*P* < 0.05, two-tailed Fisher’s exact test).

The huge majority of phosphorylation changes between sample groups involved a single interaction network according to STRING database, which included muscle contraction, glycolysis, actin polymerization, mitochondrial electron transport and stress response related phosphoproteins. This result suggests that reversible phosphorylation in a few interacting proteins can provoke rapid and extensive changes of meat proteome expected to occur in response to PSS. In addition, ACTA1 and MYLPF seem to play a pivotal role in the interaction network because they are the only two proteins mediating the interactions between structural-contractile muscle phosphoproteins and other interacting phosphoproteins. In particular, the ACTA1 protein binds to the largest number of interacting partners, which suggests that it might be a hub protein with a key role in PSS response. Most (71%) phosphorylated actin isoforms [i.e. ACTA1 (3–7)] were only identified in relatively less tender control meat. A number of studies have associated decreased beef tenderness with increasing ACTA1 phosphorylation [2, 5]. This relationship can be explained taking into account that ACTA1 phosphorylation prevents the activation of caspase 3, halting the pathway to apoptosis [2]. Li et al. [47] also reported that the phosphorylation of ACTA1 by protein kinase A prevents its degradation by μ-calpains.

The MYL2 isoforms [i.e. MYL2 (1–2), MYLPF (1–2)] showed opposite phosphorylation patterns in DFD and control meats. Thus, MYL2 (1–2) isoforms increased their phosphorylation levels in relatively less tender normal meat, which is in agreement with previous studies. Reversible phosphorylation of MYLPF regulates the myosin function and is accomplished by opposing activities of Ca^2+^/calmodulin-dependent skeletal muscle myosin light chain kinase and protein phosphatase type 1 [48–50]. In the *post*-*mortem* muscles, MYL2 phosphorylation by myosin light chain kinase is stimulated through sarcoplasmic reticulum Ca^2+^ release in a concentration-dependent manner [50, 51]. It has been reported that MYL2 phosphorylation occurs during *rigor mortis* formation and increases at 24 h *post*-*mortem* in bovine, porcine and ovine meats, which supports its possible involvement in the *rigor mortis* progress [4, 6, 7, 51]. In addition, the extent of MYLPF phosphorylation is proportionally related to increased skeletal muscle contraction force of fast-twitch fibers type IIb and tough meat [4, 52, 53]. However, two highly phosphorylated (*PR* > 90%) MYLPF (1–2) isoforms showed a contrary pattern to MYL2 (1–2) because they were identified only in more tender DFD meat samples. It suggests, therefore, that usual MYLPF phosphorylation pathways can be altered during meat ageing in response to PSS. In addition MYLPF (1–2) isoforms seem to be the most important protein-based meat biomarkers in PSS response. They achieved not only the strongest quantitative change in phosphorylation status between normal and DFD meats (*RC*-values > + 0.90), but also the most intense differential abundance at the global proteome level using the same control and DFD samples [35]. Other proteins involved in the regulation of muscle contraction (TNNT) and the polymerization of actin (CAPZ) were found to be differentially phosphorylated in DFD and normal meats. Numerous studies support the fact that TNNT is an important substrate of the proteolytic enzymes and the relationship between TNNT degradation and tenderization [54–57]. The phosphorylation of TNNT by cyclic AMP-dependent protein kinase C increases the rate of its proteolysis by μ-calpain probably due to phosphorylation-dependent dissociation of the troponin complex [58]. Huff-Lonergan et al. [59] showed that *post*-*mortem* μ-calpain-induced degradation was positively correlated with tenderness in meat samples from LT bovine muscle. Accordingly, most (83%) of the fast [i.e. TNNT3 (1–4)] and slow [i.e. TNNT1 (2)] TNNT skeletal isoforms were phosphorylated only in more tender DFD meat. CAPZA2 phosphorylation was detected only in DFD meat (*RC* = + 0.77). CAPZA2 is an alpha-2 subunit of the F-actin-capping protein that binds the barber end of actin filaments at Z-discs and blocks actin polymerization and depolymerization [60, 61]. It has been hypothesized that phosphorylation of actin capping protein subunits by protein kinase CK2 may affect the activity of the actin capping protein at the actin filaments [62].

PGM1, ENO3, UQCRC1 and CKM metabolism phosphoenzymes underwent phosphorylation changes between DFD and normal meats. PGM1, ENO3 and UQCRC1 are co-expressed phosphoenzymes that interact with MYLPF and ACTA1 phosphoproteins as shown by STRING database. PGM1 reversibly catalyzes the conversion of glucose 1-phosphate to glucose 6-phosphate in glycolysis and glycogenesis [5]. Phosphorylation of PGM1 significantly enhances its enzymatic activity in response to an increase of glycogenolysis during *post*-*mortem* metabolism [63, 64]. Anderson et al. [65] reported that more tender meats from *longissimus dorsi* bovine presented higher phosphorylated PGM1 isoforms than less tender meat samples. They hypothesized that PGM1 phosphorylation may alter the rate of conversion of glucose 1-phosphate to glucose 6-phosphate, inducing differences in the rate of pH decline. This suggests that energy demands caused by PSS response may provoke an increase of glycogenolysis, enhancing PGM1 phosphorylation in DFD meat. However, D’Alessandro et al. [2] proposed a phosphorylation-induced inhibition by preventing the kinase access to PGM1. On the other hand, all phosphorylated isoforms of ENO3 and CKM were only identified in DFD meat. In agreement with our results, higher phosphorylation levels of these enzymes were found to be positively correlated with pH increase in pork [18]. Β-enolase is a glycolytic enzyme that catalyses the conversion of 2-phospho-D-glycerate to phosphoenolpyruvate [66]. It has been shown that β-enolase phosphorylation increases phosphoenolpyruvate synthesis [66]. Accordingly, phosphorylation of ENO3 in DFD meat samples might be a response to the high energy demands induced by PSS. On the other hand, CKM catalyzes the interconversion of phosphocreatine and ADP to creatine and ATP [67]. Previous studies have shown that CKM is phosphorylated by AMP-activated protein kinase (AMPK) depending on phosphocreatine/creatine ratio and inhibits its activity [68, 69]. Ponticos et al. [68] proposed that long term muscle contraction under extreme situations reduces the phosphocreatine/creatine ratio and full activation of AMPK, ensuring that there will be sufficient ATP to sustain muscle contraction. In this line, increased muscle contraction caused by PSS could lead to increased CKM phosphorylation. Finally, UQCRC1 phosphorylation was only identified in control meat. UQCRC1 is a subunit of the cytochrome bc1 complex (or complex III) of the mitochondrial respiratory chain that transports the electrons from ubiquinol to cytochrome c [70]. The cleavage of cytochrome b-c1 by caspase 3 promotes mitochondrial disruption leading to increased cytochrome c release and apoptosis [71]. Previous studies have shown that protein phosphorylation can prevent protein cleavage by the onset of caspases and apoptosis [72–74]. Therefore, UQCRC1 phosphorylation may help understand that normal meat is less tender than DFD meat at 24 h *post*-*mortem*.

The small heat shock proteins (sHSP) HSPB1 and HSPB6 were found to be differentially phosphorylated in DFD and normal meats. HSPB1 (former name HSP27) is a phosphoprotein involved in stress response, actin stability and apoptotic signalling pathways [7, 75]. Herrera-Mendez et al. [76] have proposed that the over-abundance of HSPs at the time of programmed cellular death could have a protective function on structural proteins due to its anti-apoptotic role [76]. The over-abundance of HSPs could delay the apoptotic signaling pathway during meat aging with diverse actions such as hindering the activity of caspases and other intracellular proteolytic systems. On the contrary, decreased abundance of HSPB1 would favor the disorganization and degradation of actin which in turn would weaken the myofibrillar lattice leading to increased tenderness. Overall, the experimental evidence supports that HSPB1 concentration and actin degradation are indeed related to the mean tenderness bovine muscle [77–80]. However, the study of the type of relationship between HSPB1 abundance and tenderness provided conflicting results because the decreased abundance of HSPB1 was associated with either increased [79, 80] or decreased [77, 78] tenderness. It must be highlighted, however, that the anti-apoptotic role of HSPB1 not only depends on its concentration, but also of its phosphorylation status [75]. HSPB1 is capable of inhibiting actin polymerization at the unphosphorylated state [81], but the phosphorylation of HSPB1 following stress abolishes its actin polymerization-inhibiting activity contributing to the maintenance of actin and microfilament network stability [82, 83]. In the present study, we found that HSPB1 isoforms are unequally phosphorylated in meat samples which could contribute to explain their difference in tenderness. The effect of HSPB1 on meat tenderness could be reinforced by HSPB6 (former HSP20) phosphorylation. It has been shown that the phosphorylated isoform of HSPB6 interacts with universal adapter protein 14–3-3 inhibiting the interaction of phosphorylated cofilin with 14–3-3 that induces fragmentation and depolymerization of actin filaments [75].

## Conclusions

The present study reports pronounced phosphoproteome changes in DFD beef. Phosphoproteins with differential phosphorylation status between DFD and normal meat were involved in structural-contractile, metabolism, electron transport chain, actin polymerization and stress response related functions. Most (97%) of these phosphoproteins were only detected in either DFD or unaffected meat samples. They could therefore be candidate biomarkers of DFD meat from the LT muscle of the Rubia Gallega breed. Phosphoprotein changes were consistently associated with expected tenderness variations according to previously reported proteomic studies. It is also noteworthy that most differentially changed phosphoproteins were clustered in a single protein-protein interaction network, which can help to understand extensive meat quality variations induced by PSS in a very short period of time. Further follow-up studies are clearly necessary to assess meat phosphoproteome changes linked to the wide range of exogenous and endogenous animal factors that modulate PSS effects, different muscles and breeds, as well as the identification of phosphosites and crosstalk between kinases and phosphatases. This huge challenge will probably require the application of multi-omics technologies.

## Methods

### Sample information

Phosphoproteomic analyses were performed from four biological replicates of DFD and control (non-DFD) meat samples of the LT muscle extracted from male calves of the Rubia Gallega breed (Spain), which were previously used to assess the response of total proteome to PSS [35]. Briefly, meat samples were taken according to the usual practices in the Spanish beef industry and European Union regulations (Council Directive 93/119/EEC). Animals with a mean age of 10 months were transported from family farms to the abattoir in a time not exceeding one hour, stunned with a captive bolt, slaughtered and dressed in an accredited abattoir (Lugo, Spain). Carcasses were chilled for 24 h in a refrigerated chamber at 2 °C and relative humidity of 98%. The LT muscle was excised from the left half of each carcass, 2.5 cm thick steaks were taken at the fifth rib, vacuum-packed and transported to the laboratory under chilled conditions. Meat quality parameters distinguishing DFD and control meat were evaluated: pH at 24 h *post*-*mortem*, color in the CIELAB space [lightness (L*), redness (a*) and yellowness (b*)], water holding capacity (WHC, cooking loss), Warner-Bratzler (WB, shear force) and textural profile analysis (TPA, hardness) tests [35]. Once DFD meat samples were identified according with quality parameter values, control samples from the same farm and slaughtered the same day than DFD samples were selected for the study. Quality parameters were evaluated in meat samples from a total of 76 male calves in order to eventually obtain four biological replicates of each type of meat. Therefore, the incidence of DFD defect was 5.3%. Statistically significant differences (*P* < 0.05, Mann-Whitney U test) between DFD and control meat samples were detected for all meat quality attributes analyzed (Additional file 1: Figure S2). It can be seen that DFD samples fulfil all typical meat-quality parameter scores of DFD condition [25]. Thus, ultimate pH values in DFD and control samples were always higher and lower than 6.0, respectively. In addition, DFD meats showed darker color in the CIELAB space, higher WHC, and lower shear force (WB-test) and hardness (TPA-test) cuts. A statistically significant correlation of pH-values with L* (*P* < 0.01, *r*_*s*_ = − 0.98, *n* = 8, Spearman’s nonparametric coefficient of rank correlation), b* (*P* < 0.01, *r*_*s*_ = − 0.91, n = 8), WB (*P* < 0.01, *r*_*s*_ = − 0.93, n = 8), and TPA (*P* < 0.05, *r*_*s*_ = − 0.73, n = 8) values was detected [35], in accordance with previous observations [32, 33]. Finally, meat samples were lyophilized separately under optimal conditions [84] and subsequently frozen at − 80 °C until the time of protein extraction.

### Protein extraction and two-dimensional electrophoresis (2-DE)

Total protein extracts from lyophilized meat samples were obtained according to Franco et al. [35]. Extraction and protein purification were performed with the Clean-Up kit (GE Healthcare, Uppsala) from crude cell lysates obtained by ultrasonic disruption using a Branson digital sonifier (model S-250, Branson Ultrasonics, Danbury). Protein concentration in samples was assessed with an improved Bradford method using the CB-X protein assay kit (G-Biosciences, St. Louis) following the instructions of the manufacturer to remove interfering agents and use with a microplate reader. The bovine serum albumin (BSA) protein standard was used to determine protein concentration from calibration curves.

Proteins from lyophilized meat samples were separated by 2-DE as previously described by Franco et al. [35]. Briefly, 350 μg of each biological replicate were loaded onto an immobilized pH gradient (IPG) strip (24-cm long, pH 4–7 linear gradient, ReadyStrip IPG strips, Bio-Rad Laboratories, Hercules). First-dimension isoelectric focusing (IEF) of proteins in strip gel was performed using a PROTEAN IEF cell system (Bio-Rad Laboratories). The second dimension was run on 12% SDS-polyacrylamide gel electrophoresis (SDS-PAGE) using an Ettan DALTsix large vertical electrophoresis system (GE Healthcare).

### Phosphoprotein and total protein gel staining

Pro-Q Diamond phosphoprotein stain (Thermo Fisher Scientific, Waltham) was used as a probe for multiplex in-gel detection of phosphorylated polypeptides following the procedure described in Agrawal and Thelen [85]. The PeppermintStick™ (Thermo Fisher Scientific) phosphoprotein molecular weight standards (phosphorylated proteins: ovalbumin/45.0 kDa and β-casein/23.6 kDa; unphosphorylated proteins: β-galactosidase/116.25 kDa, bovine serum albumin/66.2 kDa, avidin/18.0 kDa and lysozyme/14.4 kDa) were used as phosphorylation controls. Phosphorylated and unphosphorylated PeppermintStick protein markers were added to meat protein extracts prior to 2-DE. Pro-Q Diamond-stained gels were post-stained for total protein density with SYPRO Ruby protein gel fluorescent stain (Lonza, Rockland) following the manufacturer’s indications.

### Image analysis

The 2-DE images from gels stained with Pro-Q Diamond and SYPRO Ruby fluorescent dyes were captured with the Gel Doc XR+ Imaging System (Bio-Rad Laboratories). Analysis of digitalized gel images was performed with PDQuest Advanced software v. 8.0.1 (Bio-Rad Laboratories). Protein volumes of detected and matched spots over biological replicates were measured following subtraction of background noise and total valid spot normalization. Automatic spot analysis by PDQuest software was manually validated. Only protein spots reproducibly detected in at the least two of four biological replicates were selected for image analyses. The observed isoelectric point (p*I*) value of protein spots was determined from their gel position relative to focused strips of linear pH gradient, whereas molecular mass markers ranging from 15 to 200 kDa (Fermentas, Ontario) were used to assess the observed molecular mass (*M*_r_). Protein fragments were identified by comparing the *M*_r_ observed on 2-DE gels with the theoretical *M*_r_ of the full-length sequence and they were excluded from further analysis.

### Mass spectrometry (MS) analysis

Protein identification was performed by MALDI–TOF and MALDI-TOF/TOF MS following the procedure described in Franco et al. [35]. In-gel proteolytic digestion of selected protein spots was performed with modified porcine trypsin (Promega, Madison) as described previously [86]. Tryptic peptides were concentrated in a SpeedVac (Thermo Fisher Scientific) and stored at − 20 °C. Dried peptide samples were dissolved in 4 μl 0.5% formic acid and subsequently mixed with an equal volume (0.5 μL) of matrix solution: 3 mg of cyano-4-hydroxycinnamic acid (CHCA) dissolved in 1 mL of 50% acetonitrile (ACN) and acidified with 0.1% trifluoroacetic acid (TFA). The resulting mixture was deposited onto a 384 Opti-TOF MALDI target plate (Applied Biosystems, Foster City) by applying the “thin layer” procedure [87]. MS data were acquired with a 4800 MALDI-TOF/TOF mass spectrometer (Applied Biosystems). Mass spectra of each sample were obtained in positive-ion reflector mode with an Nd:YAG laser operating at 355 nm, an average accumulation of 1000 laser shots and at least three trypsin autolysis peaks for internal calibration. Fragmentation of selected precursor ions was detected with a relative resolution of 300 (FWHM) and metastable suppression. Mass data were analyzed automatically using the 4000 Series Explorer Software v. 3.5 (Applied Biosystems). Combined search of peptide mass-fingerprinting (PMF) and MSMS fragment-ion spectra against the *B. taurus* UniProtKB/Swiss-Prot databases was performed with GPS Explorer Software v. 3.6 using Mascot software v. 2.1 (Matrix Science, Boston). Mascot search parameters were: one missed cleavage site allowed, precursor mass tolerance of 30 ppm, fragment mass tolerance of 0.35 Da, carbamidomethyl cysteine (CAM) as fixed modification and oxidized methionine as variable modification. All identifications and spectra were manually checked. Protein identification was validated with at least four matched peptides and statistically significant (*P*-value < 0.05) Mascot probability scores.

### Statistical analysis

The phosphorylation rate (*PR*) for each protein spot was calculated by the ratio *PR = P/T*, where *P* and *T* are the volumes of the same spot on 2-DE gels stained with Pro-Q Diamond and post-stained with SYPRO Ruby, respectively [4, 6]. Quantitative changes of *PR* between DFD and control meat samples over protein spots were estimated by the fold change (*FC*) and relative change (*RC*) coefficients [35, 44]. The coefficient *FC* was computed for each spot by *FC* = *PR*_*DFD*_/*PR*_*C*_, where *PR*_*DFD*_ and *PR*_*C*_ are the mean values of *PR* across replicate gels in DFD and control meats, respectively. *FC*-values less than one were represented as their negative reciprocal. Therefore, *FC* ranges from - ∞ to + ∞ and takes a value of + 1.0 when there is no *PR* change. The *RC* coefficient, previously used for measuring changes in protein abundance between treatments [35], was adapted to estimate changes in the status of protein phosphorylation and calculated for each spot by *RC* = *DPR*/|*DPR*_*max*_|, where *DPR* is the difference in *PR* between the two types of samples (i.e. *PR*_*DFD*_ - *PR*_*C*_) and *DPR*_*max*_ is the maximum observed value of *DPR* over spots in the study. The *RC* coefficient has the advantage that it always ranges between − 1.0 and + 1.0 and achieves a value of zero when there is no *PR* change.

Non-parametric bootstrap confidence intervals (CIs) for mean values of *PR* across four biological replicates were obtained by the bias-corrected percentile method as previously shown [35, 44]. For each observed mean of *PR*, 20000 bootstrap samples of size *N* = 4 were drawn with replacement using the random number generator of Schrage [88]. The 95% bootstrap CIs were computed from bootstrapped empirical distribution of 20000 mean values after bias correction by the percentile method using the proportion of bootstrap mean replications with a mean value lower than the observed value of the mean and the normal distribution [89]. The usual experimental type I error rate of α = 0.05 was controlled for multiple statistical comparisons on the same data using the very conservative Bonferroni adjustment. Descriptive statistics and conventional statistical tests (Mann-Whitney, Spearman’s correlation, etc.) were performed using the IBM SPSS Statistics 20 (SPSS, Chicago) software package.

Phosphoproteins were grouped into clusters by the unweighted pair-group method with arithmetic averaging (UPGMA) from the matrix of pairwise mean differences in *RC* (absolute values). The UPGMA dendrogram was generated using NTSYSpc v. 2.1 software (Applied Biostatistics, Setauket).

### Bioinformatic analysis

Functional classification of phosphoproteins grouped into biological process, molecular function and cellular component categories was carried out using high level Gene Ontology (GO) slim terms retrieved from the pre-existing GO slim generic subset (GO Consortium) by means of the Slimmer tool of AmiGO software [90]. Fine-grained information for each phosphoprotein using GO and all associated electronic and manual GO annotations provided by the GO Consortium annotation groups was retrieved by means of web-based QuickGO tool [91]. GO term enrichment analysis to find over-representation of functional annotations was performed using the FatiGO software available within the set of functional analysis tools of Babelomics 4.0 [92]. Statistically significant overrepresented functional annotations of the genes of interest with respect to the rest of the genome of *B. taurus* were determined in different databases (GO categories, KEGG and InterPro) using two-tailed Fisher’s exact tests. Adjusted *P*-values for multiple comparisons were calculated using the false discovery rate (FDR).

Map of known and predicted interaction networks for the proteins with statistically significant differences at the phosphorylation level in DFD and control samples was obtained by using the STRING v10.5 software [93, 94]. In basic settings, the “max. number of interactors to show (1st shell)” was set to zero and 1 to obtain interaction networks only between the proteins identified in our study and other proteins directly associated, respectively.

## Additional file

Additional file 1: **Table S1.** Differences in the phosphorylation rate (*PR*) over 2-DE spots between DFD and control (non-DFD) meat samples from the LT bovine muscle. **Table S2.** Differentially phosphorylated polypeptides in DFD and control (non-DFD) meat samples from the LT bovine muscle of the Rubia Gallega breed identified by MALDI-TOF and MALDI-TOF/TOF MS. **Table S3.** List of GO identifiers and terms (biological process, molecular function and cellular component) obtained by the QuickGo tool for differentially phosphorylated proteins in DFD and control meats samples from the LT bovine muscle. **Table S4.** Significantly (*P* < 0.05) overrepresented ontologies (study vs. rest of the bovine genome) in DFD and normal meats from the LT bovine muscle, after enrichment analysis by means of the FatiGo software. **Figure S1**. Phosphoprotein-phosphoprotein interaction network by means of String v10.5 considering exclusively co-expression of differentially phosphorylated proteins in DFD and control bovine meat samples. The network nodes (circles) are phosphoproteins and the edges represent co-expression associations. Threshold: 0.4. Number of interactions to show: none (1st and 2nd shell). **Figure S2**. Mean values of meat quality parameters (i.e. pH, color, water holding capacity and textural parameters measurements) over four biological replicates of DFD and control meat samples (LT bovine muscle). *Statistically significant (*P* < 0.05) differences between mean values of control and DFD meat quality parameters. (PDF 1115 kb)

## Data Availability

All data generated or analyzed during this study are included in this article and its supplementary information files.

## Abbreviations

2-DE: Two-dimensional electrophoresis
ACTA1: Actin
CAPZA2: F-actin-capping protein subunit alpha-2
CKM: Creatin kinase M-type
DFD: Dark, firm and dry
ENO3: Beta-enolase
GO: Gene Ontology
HSPB1: Heat shock protein beta-1
HSPB6: Heat shock protein beta-6
LT: *Longissimus thoracis*
MALDI-TOF: Matrix-assisted laser desorption/ionization time-of-flight
*M*_r_: Relative molecular mass
MS: Mass spectrometry
MYL2: Myosin regulatory light chain 2
MYL6B: Myosin, light chain 6B
MYLPF: Fast skeletal myosin regulatory light chain 2
PGM1: Phosphoglucomutase-1
p*I*: Isoelectric point
*PR*: Phosphorylation rate
*PR*_*C*_: Phosphorylation rate in control meat
*PR*_*DFD*_: Phosphorylation rate in DFD meat
PSE: Pale, soft and exudative
PSS: Pre-slaughter stress
*RC*: Relative change
SDS-PAGE: Sodium dodecyl sulphate-polyacrylamide gel electrophoresis
TNNT1: Slow skeletal muscle troponin T
TNNT3: Fast skeletal muscle troponin T
UQCRC1: Cytochrome b-c1 complex subunit 1
