# Supplementary material for: The first evidence of global meat phosphoproteome changes in response to pre-slaughter stress
Source: BMC Genomics. 2019 Jul 17;20:590. doi: 10.1186/s12864-019-5943-3 (PMC6637562; doi:10.1186/s12864-019-5943-3)
Supplement: Supplementary file 1 — Table S1. Differences in the phosphorylation rate (PR) over 2-DE spots between DFD and control (non-DFD) meat samples from the LT bovine muscle. Table S2. Differentially phosphorylated polypeptides in DFD and control (non-DFD) meat samples from the LT bovine muscle of the Rubia Gallega breed identified by MALDI-TOF and MALDI-TOF/TOF MS. Table S3. List of GO identifiers and terms (biological process, molecular function and cellular component) obtained by the QuickGo tool for differentially phosphorylated proteins in DFD and control meats samples from the LT bovine muscle. Table S4. Significantly (P < 0.05) overrepresented ontologies (study vs. rest of the bovine genome) in DFD and normal meats from the LT bovine muscle, after enrichment analysis by means of the FatiGo software. Figure S1. Phosphoprotein-phosphoprotein interaction network by means of String v10.5 considering exclusively co-expression of differentially phosphorylated proteins in DFD and control bovine meat samples. The network nodes (circles) are phosphoproteins and the edges represent co-expression associations. Threshold: 0.4. Number of interactions to show: none (1st and 2nd shell). Figure S2. Mean values of meat quality parameters (i.e. pH, color, water holding capacity and textural parameters measurements) over four biological replicates of DFD and control meat samples (LT bovine muscle). *Statistically significant (P < 0.05) differences between mean values of control and DFD meat quality parameters. (PDF 1115 kb) [file 12864_2019_5943_MOESM1_ESM.pdf]

Supplementary Information

BMC Genomics

The first evidence of global meat phosphoproteome changes in response to pre-slaughter stress

Ariadna Mato, Raquel Rodríguez-Vázquez, María López-Pedrouso, Susana Bravo, Daniel Franco, Carlos Zapata

Supplementary Tables 1-4

Supplementary Figures 1-2

Table S1 Differences in *PR* over 2-DE spots between DFD and control (non-DFD) meat samples from the LT bovine muscle

Spot no.[a]	DFD *PR*			Control *PR*			*P*-value[d]
	Mean (±SE)	Adjusted 95% CI (CL, CU)[b]	$P(\hat{\theta}_B \leq \hat{\theta})$[c]	Mean (±SE)	Adjusted 95% CI (CL, CU)	$P(\hat{\theta}_B \leq \hat{\theta})$	
1	0.41 ± 0.10	0.292, 0.603	0.64	0	N/A	N/A	< 0.05
2	0.42 ± 0.15	0.159, 0.815	0.56	0.45 ± 0.15	0.151, 0.615	0.63	ns
3	0.30 ± 0.48	0.204, 0.392	0.55	0.38 ± 0.10	0.084, 0.520	0.52	ns
4	0.26 ± 0.03	0.207, 0.329	0.58	0.32 ± 0.09	0.057, 0.434	0.49	ns
5	0.08 ± 0.03	0.051, 0.108	0.77	0.36 ± 0.12	0.169, 0.696	0.59	< 0.05
6	0	N/A	N/A	0.12 ± 0.06	0.060, 0.171	0.72	< 0.05
7	0.54 ± 0.21	0.206, 0.939	0.64	0	N/A	N/A	< 0.05
8	0.48 ± 0.08	0.281, 0.614	0.53	0	N/A	N/A	< 0.05
9	0.36 ± 0.10	0.153, 0.630	0.55	0	N/A	N/A	< 0.05
10	0.33 ± 0.06	0.205, 0.435	0.53	0	N/A	N/A	< 0.05
11	0.31 ± 0.03	0.238, 0.371	0.49	0	N/A	N/A	< 0.05
12	0.25 ± 0.03	0.187, 0.344	0.58	0.16 ± 0.03	0.126, 0.238	0.52	ns
13	0.22 ± 0.04	0.100, 0.293	0.56	0.21 ± 0.06	0.087, 0.367	0.55	ns
14	0.19 ± 0.06	0.062, 0.319	0.58	0.21 ± 0.04	0.105, 0.305	0.57	ns
15	0.35 ± 0.16	0.154, 0.655	0.65	0	N/A	N/A	< 0.05
16	0.31 ± 0.04	0.237, 0.379	0.57	0	N/A	N/A	< 0.05
17	0.45 ± 0.10	0.231, 0.701	0.58	0.42 ± 0.08	0.345, 0.504	0.76	ns
18	0	N/A	N/A	0.12 ± 0.04	0.067, 0.186	0.59	< 0.05
19	0	N/A	N/A	0.15 ± 0.05	0.041, 0.206	0.57	< 0.05
20	0	N/A	N/A	0.13 ± 0.02	0.105, 0.179	0.64	< 0.05

21	0	N/A	N/A	0.11 ± 0.05	0.031, 0.187	0.62	< 0.05
22	0.70 ± 0.12	0.458, 0.948	0.49	0	N/A	N/A	< 0.05
23	0.69 ± 0.08	0.505, 0.874	0.58	0	N/A	N/A	< 0.05
24	0.66 ± 0.10	0.452, 0.805	0.52	0	N/A	N/A	< 0.05
25	0.63 ± 0.06	0.447, 0.693	0.52	0	N/A	N/A	< 0.05
26	0.72 ± 0.19	0.531, 0.904	0.77	0	N/A	N/A	< 0.05
27	0	N/A	N/A	0.37 ± 0.19	0.175, 0.563	0.77	< 0.05
28	0.76 ± 0.06	0.609, 0.908	0.54	0.61 ± 0.16	0.315, 0.854	0.60	ns
29	0.61 ± 0.05	0.511, 0.721	0.55	0.78 ± 0.04	0.672, 0.871	0.51	ns
30	0	N/A	N/A	0.73 ± 0.19	0.358, 0.929	0.56	< 0.05
30A	0.71 ± 0.15	0.402, 0.879	0.50	0	N/A	N/A	< 0.05
31	0.14 ± 0.06	0.050, 0.304	0.57	0.27 ± 0.06	0.112, 0.357	0.50	ns
32	0.09 ± 0.01	0.067, 0.105	0.51	0.11 ± 0.04	0.052, 0.216	0.58	ns
33	0	N/A	N/A	0.49 ± 0.24	0.033, 0.839	0.61	< 0.05
34	0.69 ± 0.25	0.192, 0.951	0.58	0	N/A	N/A	< 0.05
35	0.79 ± 0.07	0.655, 0.925	0.55	065 ± 0.13	0.368, 0.850	0.47	ns
36	0.56 ± 0.12	0.245, 0.771	0.54	0.61± 0.18	0.421, 0.976	0.64	ns
37	0.65 ± 0.19	0.467, 0.835	0.75	0	N/A	N/A	< 0.05
38	0.54 ± 0.07	0.347, 0.687	0.50	0.45 ± 0.04	0.328, 0.529	0.55	ns
39	0.54 ± 0.03	0.347, 0.687	0.51	0.56 ± 0.13	0.308, 0.739	0.59	ns
40	0.14 ± 0.11	0.035, 0.247	0.75	0.16 ± 0.03	0.091, 0.207	0.65	ns
41	0.11 ± 0.04	0.014, 0.208	0.54	0.36 ± 0.11	0.058, 0.564	0.52	ns
42	0.07 ± 0.01	0.049, 0.098	0.59	0.08 ± 0.02	0.043, 0.104	0.55	ns
43	0.36 ± 0.06	0.234, 0.510	0.58	0.37 ± 0.11	0.257, 0.475	0.73	ns
44	0	N/A	N/A	0.46 ± 0.20	0.087, 0.776	0.60	< 0.05

45	0.49 ± 0.12	0.283, 0.733	0.59	0.48 ± 0.09	0.307, 0.616	0.57	ns
46	0	N/A	N/A	0.13 ± 0.03	0.073, 0,177	0.52	< 0.05
47	0.54 ± 0.14	0.241, 0.793	0.53	0.46 ± 0.14	0.233, 0.744	0.55	ns
48	0.55 ± 0.14	0.335, 0.802	0.58	0.62 ± 0.08	0.477, 0.819	0.51	ns
49	0.77 ± 0.04	0.669, 0.853	0.53	0.68 ± 0.05	0.529, 0.758	0.54	ns
50	0	N/A	N/A	0.09 ± 0.05	0.011, 0.194	0.67	< 0.05
51	0	N/A	N/A	0.38 ± 0.14	0.211, 0.664	0.59	< 0.05
52	0	N/A	N/A	0.37 ± 0.19	0.020, 0.764	0.54	< 0.05
53	0.93 ± 0.05	0.831, 1.000	0.55	0	N/A	N/A	< 0.05
54	0.94 ± 0.04	0.875, 1.000	0.55	0	N/A	N/A	< 0.05
55	0.86 ± 0.09	0.663, 1.000	0.53	0.93 ± 0.07	0.779, 1.000	0.64	ns
56	0.65 ± 0.20	0.248, 1.000	0.63	0.94 ± 0.02	0.889, 0.986	0.61	ns
57	0.27 ± 0.07	0.128, 0.407	0.54	0.34 ± 0.22	0.066, 1.000	0.53	ns
58	0.17 ± 0.08	0.025, 0.338	0.55	0.19 ± 0.04	0.105, 0.274	0.53	ns

[a]Gel position of assigned spots is shown in Fig. 1. [b]Simultaneous non-parametric bootstrap CIs (CL, lower bound; CU, upper bound) determined by the bias-corrected percentile method (10,000 replicates) and adjusted by the Bonferroni method. [c]The bootstrap distribution is median biased if the probability (*P*) of $(\hat{\theta}_B \leq \hat{\theta}) \neq 0.50$, where $\hat{\theta}_B$ and $\hat{\theta}$ are the bootstrap mean and the sample mean estimates, respectively. [d]ns, not statistically significant differences of *PR* between sample groups. N/A, not applicable

Table S2. Identification of differentially phosphorylated phosphoproteins in DFD and control meat samples from the LT bovine muscle of the Rubia Gallega breed by MALDI-TOF and MALDI/TOF-TOF MS

Spot no.	Type of meat	Protein identity	Abbreviation	Accession	Mascot Score	Match/Cov. (%)	Peptide	
							Sequence	$[M+H]^+$
1	DFD	Phosphoglucomutase-1	PGM1(1)	PGM1_BOVIN	415	23/45	IAAANGIGR	842.55
							LLFADGSR	878.51
							ELLSGPNR	885.52
							EAIQLIVR	941.62
							QQFDLENK	1021.52
							LYIDSYEK	1030.57
							ELEALISDR	1045.60
							NIFDFNALK	1081.62
							LSGTGSAGATIR	1090.62
							ELLSGPNRLK	1126.61
							VSQLQEKTGR	1145.59
							QEATLVVGGDGR	1201.68
							IALYETPTGWK	1278.73
							IDAMHGVVGPYVK	1401.78
							AYQDQKPGTSGLR	1420.79
							YDYEEVEAEGANK	1516.73
							FNISNGGPAPEAITDK	1630.87
							TGEHDFGAAFDGDGDR	1666.75
							SMPTSGALDRVANATK	1794.91
							IDNFEYSDPVDGSISR	1813.93
							DGLWAVLAWLSILATR	1864.94
							YDYEEVEAEGANKMMK	1923.02
							LVIGQNGILSTPAVSCIIR	2011.25
	DFD	Unidentified						
5	Control	Phosphoglucomutase-1	PGM1(2)	PGM1_BOVIN	552	20/41	DHWQK	713.28
							VSQLQEK	831.40
							IAAANGIGR	842.45
							LLFADGSR	878.41
							ELLSGPNR	885.41
							EAIQLIVR	941.51
							QQFDLENK	1021.43
							LYIDSYEK	1030.43
							ELEALISDR	1045.47
							NIFDFNALK	1081.47
							LSGTGSAGATIR	1090.49
							FFGNLMDASK	1145.45
							QEATLVVGGDGR	1201.52
							IALYETPTGWK	1278.58
							IDAMHGVVGPYVK	1401.60
							AYQDQKPGTSGLR	1420.61
							FNISNGGPAPEAITDK	1630.69
							TGEHDFGAAFDGDGDR	1666.54
							IDNFEYSDPVDGSISR	1813.68

							LSLCGEESFGTGSDHIR	1864.68
							LVIGQNGILSTPAVSCIIR	2010.95
6	Control	Cytochrome b-c1 complex	UQCRC1	QCR1_BOVIN	364	19/47	IAEVDAR	773.35
							SLLTYGR	809.39
							SGMFWLR	896.37
							NRPGNALEK	998.47
							EHTAYYIK	1024.43
							ADLTEYLSR	1067.46
							IPLAEWESR	1100.48
							FTGSQICHR	1105.44
							MVLAAAGGLEHR	1224.56
							RIPLAEWESR	1256.58
							LCTSATESEVLR	1365.56
							EVESMGAHLNAYSTR	1664.63
							LCTSATESEVLRGK	1790.72
							NALVSHLDGTTPVCEDIGR	2053.83
							HFSGLSGTYDEDAVPTLSPCR	2308.86
							VASEQSSQPTCTVGVWIDAGSR	2334.90
							AVELLADIVQNCSLEDSQIEK	2373.97
							YFYDQCPAVAGFGPIEQLPDYN[illegible]	2720.00
7	DFD	Beta-enolase	ENO3 (1)	ENOB_BOVIN	269	18/39	GVPLYR	704.44
							LGELYK	722.43
							EILDSR	732.42
							SPDDPAR	757.38
							YDLDFK	800.42
							YNQLMR	824.45
							LSVVDQEK	917.54
							TLGPALLEK	941.59
							KLSVVDQEK	1045.63
							TAIQAAGYPDK	1134.64
							NGKYDLDFK	1179.67
							FMIELDGTENK	1312.69
							GNPTVEVDLHTAK	1380.79
							IEEALGDKAVFAGR	1475.83
							VVIGMDVAASEFYR	1556.87
							VNQIGSVTESIQACK	1633.91
							AAVPSGASTGIYEALELR	1805.05
							LAMQEFMILPVGASSFR	1929.05
8	DFD	Beta-enolase	ENO3 (2)	ENOB_BOVIN	253	14/34	GVPLYR	704.44
							SPDDPAR	757.38
							YNQLMR	824.46
							IGAEVYHHLK	1166.68
							FMIELDGTENK	1392.76
							IEEALGDKAVFAGR	1475.83
							VVIGMDVAASEFYR	1556.86
							LAQSNGWGVMVSHR	1557.85
							FGANAILGVSLAVCK	1599.88

							VNQIGSVTESIQACK	1633.90
							IGAEVYHHLKGVIK	1643.94
							YNQLMRIEEALGDK	1760.04
							AAVPSGASTGIYEALELR	1805.04
							LAMQEFMILPVGASSFR	1913.06
9	DFD	Beta-enolase	ENO3 (3)	ENOB_BOVIN	269	21/48	GVPLYR	704.44
							LGELYK	722.44
							EILDSR	732.42
							SPDDPAR	757.38
							YDLDFK	800.42
							YNQLMR	824.45
							RIAQAVEK	914.59
							TLGPALLEK	941.60
							KLSVVDQEK	1045.62
							TAIQAAGYPDK	1134.63
							NGKYDLDFK	1179.67
							FMIELDGTENK	1312.66
							GNPTVEVDLHTAK	1380.77
							IEEALGDKAVFAGR	1475.83
							VVIGMDVAASEFYR	1556.85
							FGANAILGVSLAVCK	1599.88
							VNQIGSVTESIQACK	1633.91
							IGAEVYHHLKGVIK	1643.95
							VDKFMIELDGTENK	1654.89
							AAVPSGASTGIYEALELR	1805.05
							LAMQEFMILPVGASSFR	1929.07
10	DFD	Actin, alpha skeletal muscl	ACTA1 (1)	ACTS_BOVIN	474	16/62	CDIDIR	791.41
							IIAPPER	795.51
							AGFAGDDAPR	976.50
							GYSFVTTAER	1130.61
							HQGVMVGMGQK	1187.63
							AVFPSIVGRPR	1198.77
							QEYDEAGPSIVHR	1500.79
							DSYVGDEAQSKR	1514.77
							SYELPDGQVITIGNER	1791.00
							VAPEEHPTLLTEAPLNPK	1956.15
							DLYANNVMSGGTTMYPGIADR	2262.16
							MCDEDETTALVCDNGSGLVK	2294.16
							KDLYANNVMSGGTTMYPGIAD	2390.27
							LCYVALDFENEMATAASSSSLE	2552.29
							TTGIVLDSGDGVTHNVPIYEGY	3212.83
							CPETLFQPSFIGMESAGIHETTY	3220.67
							CDIDIR	791.42
							IIAPPER	795.52
							KGILTLK	852.54
							AGFAGDDAPR	976.50
							GYSFVTTAER	1130.62

							HQGVMVGMGQK	1187.64
							AVFPSIVGRPR	1198.78
11	DFD	Actin, alpha 1, skeletal mus	ACTA1 (2)	A4IFM8_BOVIN	439	15/51	QEYDEAGPSIVHR	1500.80
							IWHHTFYNELR	1515.84
							SYELPDGQVITIGNER	1791.00
							VAPEEHPTLLTEAPLNPK	1956.15
							DLYANNVMSGGTTMYPGIADR	2246.14
							KDLYANNVMSGGTTMYPGIAD	2390.26
							LCYVALDFENEMATAASSSLE	2552.27
							TTGIVLDSGDGVTHNVPIYEGY	3196.82
							DWPDAR	759.37
							IEEIFK	778.46
							FCVGLQK	851.47
							FEEILTR	907.53
							LMVEMEK	911.53
							ALTLEIYK	950.58
							RFCVGLQK	1007.59
15	DFD	Creatin kinase M-type	CKM (1)	KCRM_BOVIN	266	16/39	HGGFKPTDK	1066.60
							GYALPPHCSR	1157.61
							DLFDPIIQDR	1231.69
							GQSIDDMIPAQK	1318.69
							LSVEALNSLTGEFK	1507.79
							GGDDLDPNYVLSSR	1507.79
							LGSSEVEQVQLVVDGVK	1786.04
							GTGGVDTAAVGSVFDVSNADR	1995.05
							RGTGGVDTAAVGSVFDVSNAD	2151.14
							VISMEK	722.40
							DWPDAR	759.37
							IEEIFK	778.45
							FCVGLQK	851.48
							FEEILTR	907.53
							LMVEMEK	911.52
							ALTLEIYK	950.58
							HGGFKPTDK	986.55
							RFCVGLQK	1007.59
16	DFD	Creatin kinase M-type	CKM (2)	KCRM_BOVIN	369	20/49	GYALPPHCSR	1157.61
							PFGNTHNKHK	1179.65
							AEEEYPDLSK	1180.61
							DLFDPIIQDR	1231.69
							GGVHVKLAHLSK	1245.67
							GQSIDDMIPAQK	1318.68
							GGDDLDPNYVLSSR	1507.79
							LSVEALNSLTGEFK	1507.79
							LGSSEVEQVQLVVDGVK	1786.04
							GTGGVDTAAVGSVFDVSNADR	1995.04
							RGTGGVDTAAVGSVFDVSNAD	2151.14
							CDIDIR	791.30

18	Control	Actin, alpha, skeletal muscl	ACTA1 (3)	ACTS_BOVIN	519	11/43	IIAPPER	795.40
							AGFAGDDAPR	976.37
							GYSFVTTAER	1130.45
							HQGVMVGMGQK	1187.47
							QEYDEAGPSIVHR	1500.58
							SYELPDGQVITIGNER	1790.74
							VAPEEHPTLLTEAPLNPK	1955.86
							DLYANNVMSGGTTMYPGIADR	2277.81
							LCYVALDFENEMATAASSSLE	2551.88
							CPETLFQPSFIGMESAGIHETTY	3220.24
19	Control	Actin, alpha, skeletal muscl	ACTA1 (4)	ACTS_BOVIN	453	10/39	IIAPPER	795.40
							AGFAGDDAPR	976.37
							GYSFVTTAER	1130.45
							HQGVMVGMGQK	1187.47
							QEYDEAGPSIVHR	1500.58
							SYELPDGQVITIGNER	1790.74
							VAPEEHPTLLTEAPLNPK	1955.87
							CDEDETTALVCDNGSGLVK	2082.82
							DLYANNVMSGGTTMYPGIADR	2277.79
							LCYVALDFENEMATAASSSLE	2551.93
20	Control	Actin, alpha, skeletal muscl	ACTA1 (5)	ACTS_BOVIN	423	12/33	CDIDIR	791.32
							IIAPPER	795.42
							AGFAGDDAPR	976.39
							DLTDYLMK	1014.41
							GYSFVTTAER	1130.48
							HQGVMVGMGQK	1203.50
							QEYDEAGPSIVHR	1500.62
							GYSFVTTAEREIVR	1707.65
							SYELPDGQVITIGNER	1790.76
							VAPEEHPTLLTEAPLNPK	1955.89
							DLYANNVMSGGTTMYPGIADR	2277.85
							KDLYANNVMSGGTTMYPGIAD	2726.13
21	Control	Actin, alpha skeletal muscl	ACTA1 (6)	ACTS_BOVIN	484	12/53	CDIDIR	791.30
							IIAPPER	795.41
							GYSFVTTAER	1130.45
							HQGVMVGMGQK	1187.47
							QEYDEAGPSIVHR	1500.58
							SYELPDGQVITIGNER	1790.74
							VAPEEHPTLLTEAPLNPK	1955.87
							MCDEDETTALVCDNGSGLVK	2293.83
							DLYANNVMSGGTTMYPGIADR	2405.88
							TTGIVLDSGDGVTHNVPIYEGY	3212.30
							CPETLFQPSFIGMESAGIHETTY	3220.20
22	DFD	Troponin T, fast skeletal m	Tnnt3 (1)	TNNT3_BOVIN	66	4/8	YDITNLR	894.49
							VDFDDIQK	979.50
							FEYGEKLK	1093.56
							VDFDDIQKK	1107.57

23	DFD	Troponin T fast skeletal mu	Tnnt3 (2)	Q8MKH7_BOVIN	62	4/13	YDITNLR	894.48
							VDFDDIQK	979.49
							ALSSMGANYSSYLAK	1578.77
							ALSSMGANYSSYLAKADQK	2261.17
24	DFD	Troponin T, fast skeletal m	Tnnt3 (3)	TNNT3_BOVIN	145	7/13	RAEDDLK	846.46
							YDITNLR	894.47
							VDFDDIQK	979.48
							VDFDDIQKK	1107.54
							QKYDITNLR	1150.63
							LTAPKIPEGEK	1182.61
							IPEGEKVDFDDIQK	1632.81
25	DFD	Troponin T, fast skeletal m	Tnnt3 (4)	TNNT3_BOVIN	60	5/13	YDITNLR	894.51
							VDFDDIQK	979.51
							VDFDDIQKK	1107.59
							ALSSMGANYSSYLAK	1578.81
							IPEGEKVDFDDIQK	1632.87
26	DFD	F-actin-capping protein sub	CAPZA2	CAZA2_BOVIN	74	5/16	QLPVTR	713.44
							TSVETALR	876.49
							IGKEMQNA	890.48
							LLLNNDNLLR	1197.72
							MADLEEQLSDEEKVR	1791.84
27	Control	Actin, alpha skeletal muscl	ACTA1 (7)	ACTS_BOVIN	300	7/23	IIAPPER	795.39
							GYSFVTTAER	1130.46
							QEYDEAGPSIVHR	1500.60
							GYSFVTTAEREIVR	1707.63
							SYELPDGQVITIGNER	1790.73
							VAPEEHPTLLTEAPLNPK	1955.85
							CDEDETTALVCDNGSGLVK	2082.80
30	Control	Troponin T, slow skeletal n	TNNT1 (1)	TNNT1_BOVIN	109	6/20	IPEGER	700.30
							FDLMAK	740.30
							VDFDDIHR	1016.40
							ISHAQKFR	1066.42
							YEINVLYNR	1183.51
							DLLELQTLIDVHFEQR	1968.85
30A	DFD	Troponin T, slow skeletal n	TNNT1 (2)	TNNT1_BOVIN	71	5/14	IPEGER	700.38
							QTGREMK	865.53
							VDFDDIHR	1016.52
							ISHAQKFR	1066.54
							YEINVLYNR	1183.66
33	Control	Heat shock protein beta-1	HSPB1 (1)	HSPB1_BOVIN	230	5/36	LFDQAFGLPR	1163.48
							ALPAAAIEGPAYNR	1413.57
							VSLDVNHFAPEELTVK	1797.69
							SATQSAEITIPVTFQAR	1819.72
							AQLGGPEAGKSEQPENK	1819.72
34	DFD	Heat shock protein beta-1	HSPB1 (2)	E1BEL7_BOVIN	159	3/20	LFDQAFGLPR	1163.60
							ALPAAAIEGPAYNR	1413.71
							SATQSAEITIPVTFQAR	1819.91

37	DFD	Heat shock protein beta-1	HSPB1 (3)	E1BEL7_BOVIN	163	4/27	LFDQAFGLPR	1163.60
							ALPAAAIEGPAYNR	1413.72
							QLSSGVSEIQQTADR	1618.79
							SATQSAEITIPVTFQAR	1819.92
44	Control	Myosin, light chain 6B, alk	MYL6B (1)	Q148H2_BOVIN	109	6/26	VMGAELR	791.34
							EAFELYDR	1042.41
							IQEPPIDLSK	1139.52
							GSYQDYLEGLR	1300.49
							ALGQNPTNAEVLR	1382.62
							IQEPPIDLSKVVIEFNK	1968.88
46	Control	Myosin, light chain 6B, alk	MYL6B (2)	Q148H2_BOVIN	329	15/58	VMGAELR	791.34
							VVIEFNK	848.41
							DQLEEFK	908.37
							HVLTTLGER	1025.48
							EAFELYDR	1042.39
							VFDKEQNGK	1064.45
							IQEPPIDLSK	1139.53
							GSYQDYLEGLR	1300.50
							IQFSQCGDVMR	1356.48
							ALGQNPTNAEVLR	1382.62
							VDFETFLPMLQAVAK	1724.72
							VVIEFNKDQLEEFK	1737.75
							VELPSLIPVILEKPAK	1745.91
							RVDFETFLPMLQAVAK	1880.82
							DQLEEFKEAFELYDR	1931.74
50	Control	Heat shock protein beta-6	HSPB6	HSPB6_BOVIN	146	3/39	HFSPEEIAVK	1156.48
							ASAPLPGLSAPGR	1193.54
							LPPGVDPAAVTSALSPE	3912.67
51	Control	Myosin regulatory light cha	MYL2 (1)	MLRV_BOVIN	399	10/65	VFDPEGK	791.31
							DTFAALGR	850.35
							EMLTTQAER	1094.40
							NEEIDEMLK	1136.38
							DGFIDKNDLR	1192.46
							EAFTIMDQNR	1240.43
							GADPEETILNAFK	1404.53
							EAPGPINFTVFLQMFGE	2040.76
							EEIDQMFAAFPPDVTGN	2415.82
							FSKEEIDQMFAAFPPDV	2778.03
52	Control	Myosin regulatory light cha	MYL2 (2)	F1ME15_BOVIN	380	11/76	VFDPEGK	791.28
							DTFAALGR	850.32
							EMLTTQAER	1094.36
							NEEIDEMLK	1136.35
							DGFIDKNDLR	1192.43
							EAFTIMDQNR	1240.39
							GADPEETILNAFK	1404.50
							EAPGPINFTVFLQMFGEK	2040.71
							EEIDQMFAAFPPDVTGNL	2415.77

							AEGANSNVFSMFEQTQI	2420.78
							RAEGANSNVFSMFEQTQI	2576.86
53	DFD	Myosin regulatory light cha	MYLPF (1)	MLRS_BOVIN	363	10/61	DTFAAMGR	868.35
							FSQEEIK	880.39
							DGIIDKEDLR	1173.55
							EAFTVIDQNR	1192.53
							GADPEDVITGAFK	1319.57
							FLEELLTTQCDR	1524.65
							LKGADPEDVITGAFK	1560.73
							EASGPINFTVFLNMFGEK	2016.83
							AAAEGGSSSVFSMFDQTQIQEFI	2480.95
							RAAAEGGSSSVFSMFDQTQIQEI	2716.87
54	DFD	Myosin regulatory light cha	MYLPF (2)	MLRS_BOVIN	88	4/23	DTFAAMGR	884.35
							DGIIDKEDLR	1173.55
							EAFTVIDQNR	1192.53
							FLEELLTTQCDR	1524.64

Table S3 List of GO identifiers and terms (biological process, molecular function and cellular component) obtained by the QuickGo tool for differentially phosphorylated proteins in DFD and control meats

Protein name	Abbrev.	UniprotKB accession number	Ensemble gene accession number	GO Identifier and GO term name
Phosphoglucomutase-1	PGM1	Q08DP0	ENSBTAG00000019011	**Process:** GO:0005975 (carbohydrate metabolic process) GO:0005978 (glycogen biosynthetic process) GO:0006006 (glucose metabolic process) GO:0019388 (galactose catabolic process) GO:0071704 (organic substance metabolic process) **Function:** GO:0000287 (magnesium ion binding) GO:0004614 (phosphoglucomutase activity) GO:0004614 (phosphoglucomutase activity) GO:0016868 (intramolecular transferase activity, phosphotransferases) GO:0046872 (metal ion binding) **Component:** GO:0005737 (cytoplasm) GO:0005829 (cytosol)
Cytochrome b-c1 complex subunit 1, mitocondrial	UQCRC1	P31800	ENSBTAG00000019096	**Process**: GO:0006122 (mitochondrial electron transport, ubiquinol to cytochrome c) GO:0009060 (aerobic respiration) GO:0016485 (protein processing) GO:0055114 (oxidation-reduction process) **Function**: GO:0003824 (catalytic activity) GO:0004222 (metalloendopeptidase activity) GO:0008270 (zinc

				ion binding) GO:0046872 (metal ion binding) **Component**: GO:0005739 (mitochondrion) GO:0005743 (mitochondrial inner membrane) GO:0005750 (mitochondrial respiratory chain complex III) GO:0016020 (membrane) GO:0070469 (respiratory chain)
Beta-enolase	ENO3	Q3ZC09	ENSBTAG00000005534	**Process**: GO:0006096 (glycolytic process) **Function**: GO:0000287 (magnesium ion binding) GO:0004634 (phosphopyruvate hydratase activity) GO:0016829 (lyase activity) GO:0046872 (metal ion binding) **Component**: GO:0000015 (phosphopyruvate hydratase complex) GO:0005615 (extracellular space) GO:0005737 (cytoplasm) GO:0005886 (plasma membrane) GO:0070062 (extracellular exosome)
Actin, alpha skeletal muscle	ACTA1	P68138	ENSBTAG00000046332	**Process**: GO:0010628 (positive regulation of gene expression) GO:0030240 (skeletal muscle thin filament assembly) GO:0048741 (skeletal muscle fiber development) GO:0090131 (mesenchyme migration) **Function**: GO:0000166 (nucleotide binding) GO:0005524 (ATP binding) **Component**: O:0001725 stress fiber GO:0005615 (extracellular space) GO:0005737 (cytoplasm) GO:0005856 (cytoskeleton) GO:0005865 (striated

				muscle thin filament) GO:0005884 (actin filament) GO:0015629 (actin cytoskeleton) GO:0030017 (sarcomere) GO:0030027 (lamellipodium) GO:0030175 (filopodium) GO:0044297 (cell body) GO:0070062 (extracellular exosome) GO:0072562 (blood microparticle)
Creatin kinase M-type	CKM	Q9XSC6	ENSBTAG00000013921	**Process**: GO:0009408 (response to heat) GO:0016310 (phosphorylation) GO:0046314 (phosphocreatine biosynthetic process) **Function:** GO:0000166 (nucleotide binding) GO:0003824 (catalytic activity) GO:0004111 (creatine kinase activity) GO:0004111 (creatine kinase activity) GO:0016301 (kinase activity) GO:0016740 (transferase activity) GO:0016772 (transferase activity, transferring phosphorus-containing groups) **Component:** GO:0005615 (extracellular space) GO:0005737 (cytoplasm)
Troponin T, fast skeletal muscle	TNNT3	Q8MKI3	ENSBTAG00000022158	**Process**: GO:0003009 (skeletal muscle contraction) GO:0006937 (regulation of muscle contraction) GO:0006942 (regulation of striated muscle contraction) GO:0043462 (regulation of ATPase activity) **Function**: GO:0003779 (actin binding) GO:0005523 (tropomyosin binding) GO:0030172 (troponin C binding) GO:0030899 (calcium-dependent ATPase

				activity) GO:0031013 (troponin I binding) GO:0048306 (calcium-dependent protein binding) **Component**: GO:0005861 (troponin complex)
F-actin-capping protein subunit alpha-2	CAPZA2	Q5E997	ENSBTAG00000004072	**Process:** GO:0051016 (barbed-end actin filament capping) GO:0051693 (actin filament capping)**Function**: GO:0003779 (actin binding) **Component:** GO:0005903 (brush border) GO:0008290 (F-actin capping protein complex) GO:0016020 (membrane) GO:0030863 (cortical cytoskeleton) GO:0070062 (extracellular exosome)
Troponin T, slow skeletal muscle	TNNT1	Q8MKH6	ENSBTAG00000006419	**Process:** GO:0003009 (skeletal muscle contraction) GO:0006937 (regulation of muscle contraction) GO:0014883 (transition between fast and slow fiber) GO:0045932 (negative regulation of muscle contraction) **Function**: GO:0005523 (tropomyosin binding) **Component:** GO:0005861 (troponin complex)
Heat shock protein beta-1	HSPB1	Q3T149	ENSBTAG00000011969	**Component**: GO:0005634 (nucleus) GO:0005737 (cytoplasm) GO:0005819 (spindle) GO:0005856 (cytoskeleton)
Myosin, light chain 6B, alkali, smooth muscle and non-muscle	MYL6B	Q148H2	ENSBTAG00000031217	**Function**: GO:0005509 (calcium ion binding) **Component:** GO:0070062 (extracellular exosome)

Heat shock protein beta-6	HSPB6	Q148F8	ENSBTAG00000018598	**Function**: GO:0005212 (structural constituent of eye lens) GO:0042803 (protein homodimerization activity) **Component:** GO:0005634 (nucleus) GO:0005737 (cytoplasm)
Myosin regulatory light chain 2, ventricular/cardiac muscle isoform	MYL2	Q3SZE5	ENSBTAG00000018369	**Process:** GO:0002026 (regulation of the force of heart contraction) GO:0007507 (heart development) GO:0060047 (heart contraction) GO:0098735 (positive regulation of the force of heart contraction) **Function** GO:0005509 (calcium ion binding) GO:0046872 (metal ion binding) **Component:** GO:0005737 (cytoplasm) GO:0016459 (myosin complex) GO:0031672 (A band)
Myosin regulatory light chain 2, skeletal muscle isoform	MYLPF	Q0P571	ENSBTAG00000021218	**Process:** GO:0007519 (skeletal muscle tissue development) **Function**: GO:0005509 (calcium ion binding) GO:0008307 (structural constituent of muscle) GO:0046872 (metal ion binding) **Component:** GO:0005765 (lysosomal membrane) GO:0016459 (myosin complex)

Table S4 Significantly (P < 0.05) overrepresented ontologies (present study versus rest of the bovine genome) in DFD and control meat after enrichment analysis by means of the FatiGo software

Database/GO term	Term size	Term annotation (%)/ study vs genome	Protein (abbrev.)	Odds ratio ($\log_e$)	P-value[a]	Adjusted P-value[b]
KEGG/						
bta01100	732	30.8 vs 3.4	PGM1, UQCRC1, ENO3, CKM	2.52	0.001	0.040
bta00010	42	15.4 vs 0.3	PGM1, ENO3	4.56	0.0003	0.037
bta04260	48	15.4 vs 0.2	UQCRC1, MYL2	4.42	0.0004	0.037
bta04530	75	15.4 vs 0.4	MYL2, MYLPF	3.96	0.001	0.040
bta04810	97	15.4 vs 0.5	MYL2, MYLPF	3.69	0.002	0.042
bta04510	90	15.4 vs 0.4	MYL2, MYLPF	3.77	0.001	0.042
bta04670	79	15.4 vs 0.4	MYL2, MYLPF	3.90	0.001	0.040
InterPro/						
Troponin (IPR001978)	5	15.4 vs 0.01	TNNT3, TNNT1	7.15	0.000004	0.007
Alpha crystallin/heat shock protein (IPR001436)	9	15.4 vs 0.03	HSPB1, HSPB6	6.30	0.00001	0.012

Heat shock protein Hsp20 (IPR002068)	11	15.4 vs 0.04	HSPB1, HSPB6	6.05	0.00002	0.012
GO cellular component/						
actin cytoskeleton (GO:0015629)	236	23.1 vs 1.1	CAPZA2, TNNT1, MYL2	3.29	0.0004	0.042
myofibril (GO:0030016)	59	15.4 vs 0.3	TNNT1, HSPB1	4.20	0.0006	0.042
contractile fiber (GO:0043292)	64	15.4 vs 0.3	TNNT1, HSPB1	4.12	0.0007	0.042

[a]Probability by two-tailed Fisher probability test. [b]Adjusted *P*-values for multiple testing via false discovery rate (FDR) method. No statistically significant ($P > 0.05$) terms were found for GO biological process and GO molecular function categories

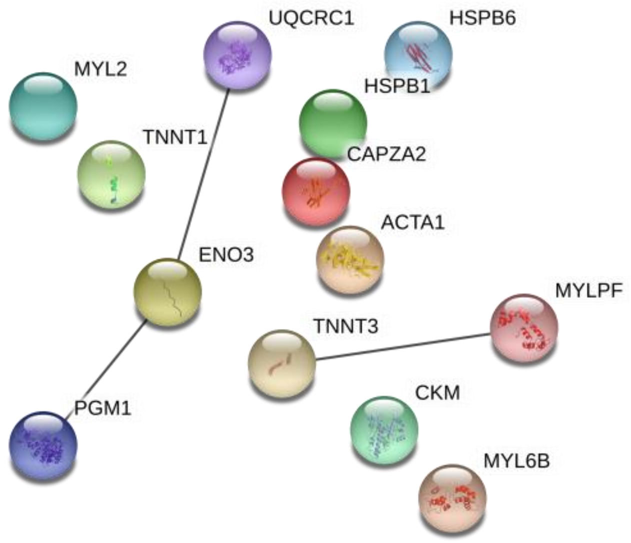

Fig. S1. Graph of co-expression networks of differentially phosphorylated proteins in DFD and control bovine meat according to STRING confidence view. The network nodes (circles) are phosphoproteins and the edges represent known or predicted functional associations (Settings: number of interactions to show, none in the first and second shell; threshold: 0.4, medium confidence interval)

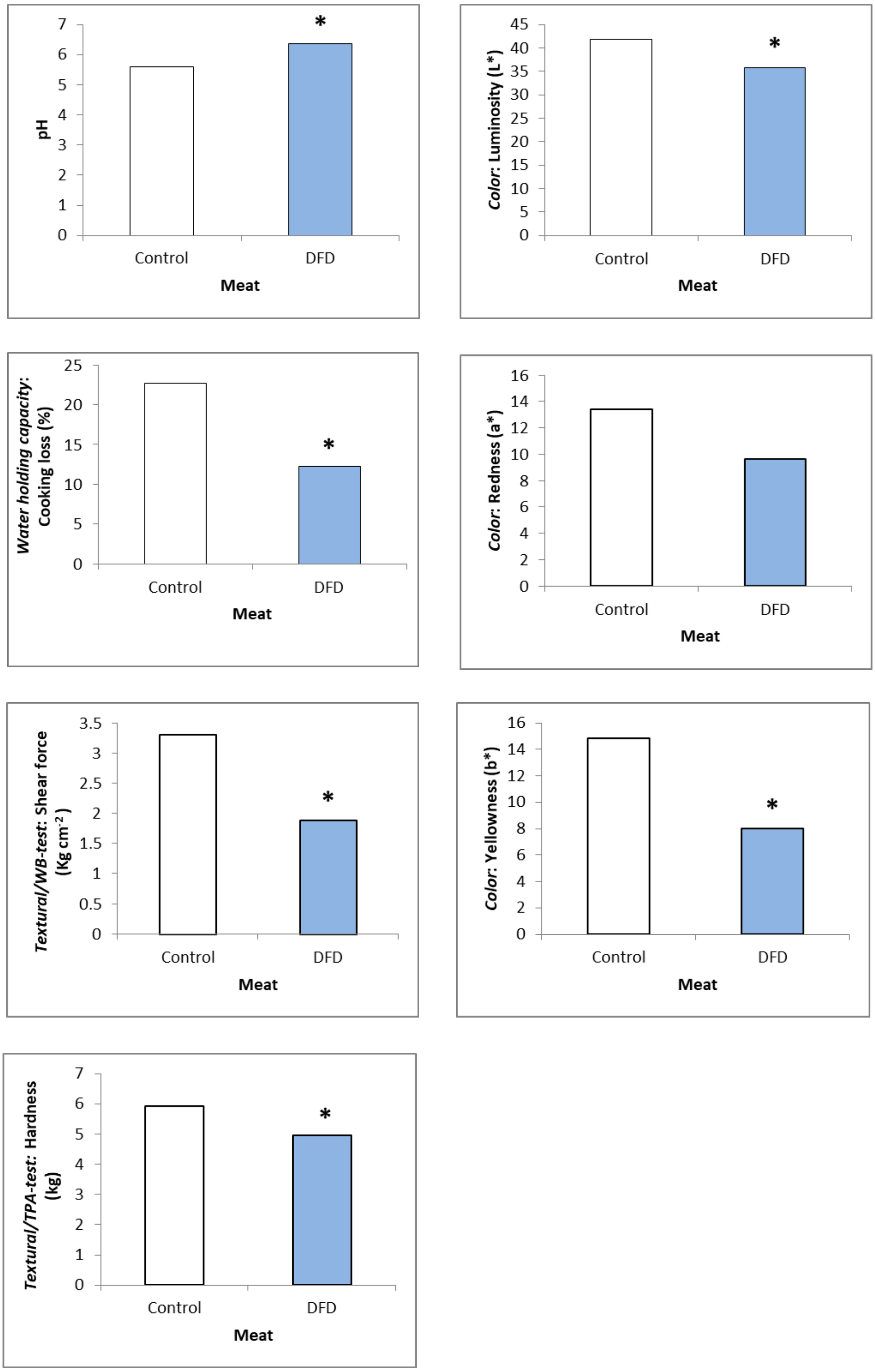

Figure S2 Mean values of quality parameters (pH, color, water holding capacity and textural parameters) over four biological replicates of DFD and control bovine meat from *longissimus thoracis* muscle [35]. Asterisks indicate statistically significant differences (*P*-value < 0.05) between sample groups by the Mann-Whitney U test.
